# Fermented Fruits, Vegetables, and Legumes in Metabolic Syndrome: From Traditional Use to Functional Foods and Medical Applications

**DOI:** 10.3390/nu17121989

**Published:** 2025-06-12

**Authors:** Karolina Bernacka, Tomasz Sozański, Alicja Z. Kucharska

**Affiliations:** 1 Department of Fruit, Vegetable and Plant Nutraceutical Technology, Wrocław University of Environmental and Life Sciences, Chełmońskiego 37, 51-630 Wrocław, Poland; alicja.kucharska@upwr.edu.pl; 2 Department of Preclinical Sciences, Pharmacology and Medical Diagnostics, Wrocław University of Science and Technology, Hoene-Wrońskiego 13 c, 58-376 Wrocław, Poland; tomasz.sozanski@pwr.edu.pl

**Keywords:** fermentation, microbial metabolites, table olives, kimchi, natto, cheonggukjang

## Abstract

Fermentation has been used for centuries to preserve food and to obtain products with new, attractive sensory characteristics. Fermented products are a source of dietary fiber, vitamins, bioactive compounds, and probiotic bacteria with health-promoting properties. This review provides a comprehensive overview of the effects of fermented fruits, vegetables, and legumes on metabolic disturbances characterizing metabolic syndrome (MetS). Furthermore, the chemical composition, microbial communities, and molecular mechanisms of action of fermented plant foods are discussed. Fermented fruits and vegetables, including table olives, caper fruits, and kimchi, contain polyphenols and probiotic bacteria, which are beneficial in terms of obesity and impaired glucose and lipid metabolism. Fermented legumes are a valuable source of bioactive peptides and isoflavone aglycones. Among fermented soybean products, natto stands out due to the presence of *γ*-polyglutamic acid, which improves glucose tolerance and the lipid profile, and nattokinase, an enzyme that acts as an angiotensin-converting enzyme inhibitor. Potential future studies focused on developing functional fermented foods and easy-to-use supplements based on fermented plant products are suggested.

## 1. Introduction

Metabolic syndrome (MetS) is a cluster of comorbid metabolic disorders that include visceral adiposity, atherogenic dyslipidemia, insulin resistance, and hypertension. Untreated MetS is associated with an increased risk of diabetes and cardiovascular disease (CVD), the main cause of death worldwide [1]. Today, MetS is also known to be associated with a higher risk of non-alcoholic fatty liver disease, neurodegenerative diseases, cancer, and gut microbial dysbiosis [2,3].

All dysregulations included in the MetS definition are strongly related to lifestyle. Some modifiable factors, such as an imbalance between energy intake and expenditure, unhealthy dietary patterns, sedentary lifestyle, poor sleeping habits, and stress, underlie metabolic disturbances [4]. The prevention and incidence of MetS are associated strongly with dietary habits. One of the most beneficial dietary patterns in MetS prevention and treatment is the Mediterranean diet, which is characterized by a high intake of plant-based foods: fruits and vegetables, together with olive oil, whole grains, legumes, and nuts [2].

Plant food contains a wide range of bioactive compounds, including polyphenols, vitamins, phytosterols, biogenic amines, and biologically active proteins, which are beneficial to human health [5]. In many cultures, some plant raw materials undergo fermentation to obtain desirable organoleptic attributes (such as flavor, smell, and texture) and an extended shelf life [6,7,8]. Fermentation is considered to be a process that can affect bioactive compounds both favorably and adversely [9]. Biologically active compounds, such as betalains, may degrade during fermentation. However, the breakdown of complex phenolic compounds can lead to the formation of simpler molecules (e.g., aglycones), which are often characterized by higher bioactivity [9].

During the fermentation, a breakdown of plant cell walls occurs, which promotes the liberation of phytocompounds. Degradation of complex polyphenols leads to an increase in total phenolic content (TPC) [10] and, consequently, an increase in the antioxidant capacity of the products [11]. During fermentation, the counteraction of toxic, anti-nutritive, and allergenic compounds takes place, which is particularly significant in the processing of legumes [12,13]. Fermented plant-based foods also contain microorganisms, including probiotic bacteria. Among probiotic bacteria, an important group occurring in fermented foods is lactic acid bacteria (LAB), which is responsible for lactic fermentation in kimchi, sauerkraut, table olives, and many others [6]. As a consequence of fermentation in fermented food, metabolites called ‘postbiotics’ occur [14]. In many cases, fermentation leads to an increase in vitamins (such as thiamine, riboflavin, cobalamin, and folic acid) [6], antioxidant polysaccharides, and antioxidant peptides, which originate from microbial hydrolysis and biotransformation [9].

This work discusses the potential use of fermented fruits, vegetables, and legumes as functional foods that can be applied in the prevention and alleviation of metabolic disturbances. It primarily focuses on the health-promoting properties in the context of MetS, as well as on the bioactive compounds and microbial communities of fermented foods. Furthermore, it discusses the potential mechanisms of the favorable effects of fermented products, their constituents, and the probiotics included in them on metabolic health. Finally, we present an overview of the recent trends in fermentation and future perspectives for the application of fermented fruits, vegetables, and legumes in MetS management.

## 2. Selection of Relevant Publications

A literature search was performed manually in the Google Scholar, Scopus, and PubMed databases, with a combination of “metabolic syndrome”, “diabetes”, “lipid profile”, “microbial community”, “chemical composition”, and product names such as “kimchi”, “sauerkraut”, “table olives”, “natto”, and others. The review included papers from the last 10 years, as well as papers published before that period that concerned animal or human studies. Because the nomenclature of bacteria has changed in the last few years, in the case of papers that use the old names, the updated nomenclature is given in parentheses.

## 3. Fermentation

**Figure 1:**
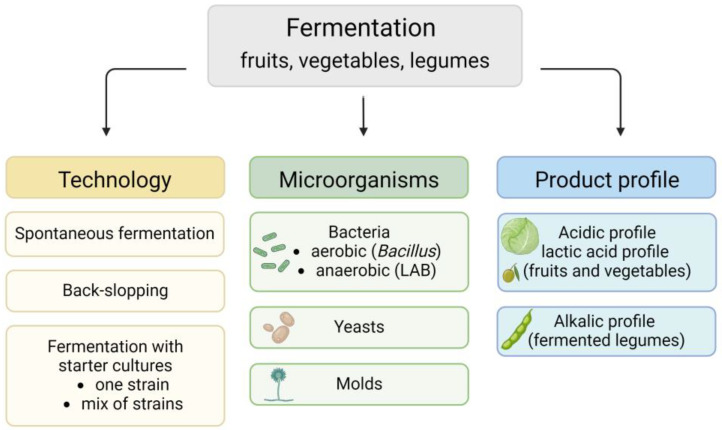
Fermentation classification according to technology, type of microorganisms carrying out the fermentation, and product profile.

Fermentation is defined as a process in which the metabolic activity and growth of bacteria, yeasts, and molds are used to preserve food. During fermentation, microorganisms convert carbohydrates into simpler substances such as carbon dioxide, alcohol, organic acids, and other compounds [6]. Fermentation can be classified according to the technology, type of microorganisms carrying out the fermentation, and product profile (acidic, alkalic, or alcoholic) (Figure 1). According to technology, fermentation can be divided into spontaneous fermentation, fermentation with starter cultures, and back-slopping. Traditional (spontaneous) fermentation occurs in rural regions and is still applied in industry for some products, such as sauerkraut. The microbiota in spontaneous fermentation depends on microorganisms present in raw products, the environment, and equipment. Another characteristic feature of the spontaneous process is the succession of microorganisms. LAB usually occur in the primary stage of fermentation. In the final stage of fermentation, the number of LAB decreases and yeast appears. Yeast may form a white film on the surface of the product, which is associated with a deterioration of its quality [8,15,16,17]. A more predictable type of fermentation technology is back-slopping, where a portion of a product from a previously fermented product is used to inoculate a new batch. Nowadays, the most favorable method seems to be controlled fermentation with selected starter cultures (containing one or several strains) [8]. Controlled fermentation provides a short time of fermentation, reduction in spoilage, and high safety, and allows one to obtain a product with desirable organoleptic or probiotic properties [8,15].

The profile of fermented food is related to the microbial communities. One of the most common groups of bacteria carrying out the fermentation is LAB. LAB are non-spore-forming, nonaerobic, or aerotolerant, Gram-positive bacteria that conduct the fermentation of sugar to lactic acid, which is the major end-product of the fermentation. LAB possess a GRAS status (Generally Recognized as Safe), which allows their use as probiotics and starter strains [18]. Pickles produced by lactic acid fermentation are characterized by a low pH (acidic profile) and sour taste. The most popular lactic acid-fermented foods are sauerkraut, kimchi, and table olives [19].

Legumes and legume-based Asian and African products are mostly fermented by *Bacillus*. *Bacillus* is an aerobic, Gram-positive, heat-resistant, and spore-forming bacterium [20,21]. The most dominant *Bacillus* species occurring in fermented food is *Bacillus* subtilis, which, as a LAB, possesses the GRAS status. *Bacillus* shows proteolytic, amylolytic, and lipolytic activities. In the first stage of fermentation, *Bacillus* ferments carbohydrates to acids, and the pH slightly rises. This is followed by proteolysis by bacterial enzymes. Proteolysis leads to the production of alkaline compounds (ammonia and amines) through peptides and amino acids, which consequently leads to an increase in pH to about 8–10 (alkaline profile) [20,22]. Moreover, some *Bacillus* species produce an amino acid polymer, which gives a characteristic sticky texture to the fermented products [19].

Yeast is also involved in the fermentation of vegetables and legumes, although they play a secondary role. Yeasts convert sugar into alcohol, limit the growth of mycotoxin-producing molds, and exhibit enzymatic activity. In LAB-fermented foods, yeast enzymes break down the phenolic compounds responsible for inhibiting the growth of LAB. Products with an alcoholic fermentation profile, mainly conducted by yeast, include alcoholic beverages (beer, wine, vodka) and sourdough [21,22].

Some fermented foods and beverages are made with the participation of filamentous molds. Molds are characterized by their ability to produce enzymes (*α*-amylase, *β*-galactosidase, cellulase, hemicellulose, and lipases) and degrade anti-nutritional compounds. An example of a food obtained by mold fermentation is *Rhizopus*-fermented soybeans [19,21].

## 4. Fermented Fruits

The chemical composition, microbial communities, and health benefits of selected fermented fruits are presented below.

### 4.1. Table Olives

Table olives are fermented fruits of the olive tree (*Olea europaea* L.). Olives and olive oil are historically a component of the diet of people living in the Mediterranean regions. In 2010, olives were added to the Mediterranean Diet Healthy Eating Pyramid as a product that should be consumed on a daily basis due to its content of biologically active compounds, fatty acids (FA), and dietary fiber [23,24]. The production of table olives is constantly increasing. Currently, the main table olive producers are Mediterranean countries (Italy, Greece, Spain, and Portugal), as well as Turkey, Egypt, Syria, and Morocco [24]. To obtain an edible product from a bitter drupe, three main ways of processing are applied. They are called ‘Greek natural’, ‘green Spanish’, and ‘California-style black ripe’ methods.

- Greek olives are harvested in the purple stage of maturation, debittered by long soaking in brine (up to 1 year), and then fermented. To correct the color losses caused by the diffusion of anthocyanins, ferrous gluconate can be added. The final product is characterized by a sour, fermented, and salty flavor and a low pH (~4).
- Green Spanish olives are harvested in a green stage of maturation, debittered by lye treatment, and fermented in brine for up to 7 months. The ready-to-go product remains green and has a sensory profile and pH similar to natural Greek olives.
- California-style olives are obtained by an artificial method. First, the olives in a green stage of maturation undergo a debittering process by lye treatment. To obtain a black-colored product, the fruit is then oxidized by air. Lye treatment and oxidation are repeated several times, and then ferrous gluconate is added to fix the black color of the olives. The debittering process lasts only one week, and the fermentation process is omitted. The final product is characterized by an earthy, soapy, and buttery taste, a high pH (5.8–7.9), and may contain acrylamide.

As this review is focused on fermented products, in the following paragraphs, California-style olives are omitted [25].

#### 4.1.1. Nutrients and Phytocompounds

The crucial macronutrients in table olives are lipids. The amount of total fat in olives reaches values of up to 28 g/100 g of the edible portion. The most prevalent fatty acid is oleic acid, a monounsaturated fatty acid (MUFA). Olives are also a source of sterols (20–30 mg/100 g of edible portion) and fatty alcohols [24]. The bioactive compounds present in table olive fruits are secoiridoids (oleuropein, ligstroside) and phenolic compounds, including phenolic alcohols (tyrosol, hydroxytyrosol), flavonols (rutin), flavones (luteolin, apigenin glycosides), a hydroxycinnamic acid derivative (verbascoside), lignans (pinoresinol), and phenolic acids [26]. Olives also contain non-phenolic compounds belonging to triterpenic acids. The detailed chemical composition of table olives is presented in Table 1.

During fermentation, the amount of oleuropein decreases. Bacterial hydrolysis of oleuropein occurs in two steps. First, *β*-glucosidase, produced by bacteria, such as *Lactobacillus pentosus* (currently *Lactiplantibacillus pentosus*), hydrolyzes oleuropein to glucose and aglycone. The aglycone is transformed to elenolic acid and hydroxytyrosol by an esterase, another enzyme produced by LAB [23,61]. As a result, the content of hydroxytyrosol (HT) rose from 273.43 μg/g fresh tissue in fresh olives to 367.83 or 333.96 μg/g in Greek-style and Spanish-style olives, respectively [62]. The bitter compounds present in olives can also be hydrolyzed by lye treatment. Lye penetrates the flesh and hydrolyzes oleuropein and ligstroside to non-bitter compounds such as tyrosol and HT [26]. The content of bioactive compounds is closely related to the manner of olive processing. Greek-style olives contain more phenolic compounds than Spanish-style olives [63].

#### 4.1.2. Microbiota

Fermented olives contain numerous LAB species, including *Lactobacillus*, *Leuconostoc*, *Lactococcus*, *Streptococcus*, *Pediococcus*, *Weissella*, and *Enterococcus* [23,27,28,29,30]. The most frequent species in olives are *Lactobacillus plantarum* (currently *Lactiplantibacillus plantarum*) and *L. pentosus*, followed by *Lactobacillus brevis* (currently *Levilactobacillus brevis*), *Lactobacillus coryniformis* (currently *Loigolactobacillus coryniformis*), *Lactobacillus paraplantarum* (currently *Lactobacillus paraplantarum*), and *Leuconostoc mesenteroides* [27]. The bacterial community can be affected by lye treatment. Lye can influence the bacteria present both in the brine and on the surface of the fruit and inhibit desirable bacterial growth by pH changes [64]. On the other hand, water-rinsing following lye treatment enables the removal of oleuropein and some phenolic compounds, which limit LAB growth [25]. Apart from LAB, yeasts are important for olive fermentation. Yeasts promote the degradation of some phenolic compounds, which inhibit LAB growth [27]. The detailed characteristics of the microbial community of table olives are shown in Table 1.

Despite the numerous advantages of using starter strains, olives are still produced by spontaneous fermentation. In the case of olives, the use of starter strains may have additional benefits because some strains may produce *β*-glucosidase, which is related to oleuropein degradation and, as a consequence, lower bitterness [27,65,66]. The debittering effect can also be achieved by using a combined starter culture, including LAB (*L. plantarum*) and yeasts (*Saccharomyces cerevisiae*) [67].

#### 4.1.3. The Relevance of Table Olives in the Context of MetS

Oleuropein, which is considered the most pro-health compound in fresh olives, undergoes hydrolysis to HT during fruit ripening, olive processing, and storage. After ingestion, the remaining amount of oleuropein undergoes hydrolysis to HT by enzymes produced by gut microbiota [68]. As a result, HT remains the most important compound responsible for the health benefits of table olives. It is worth noting that the amount of HT in table olives (2–114 mg/100 g) [24] is higher than that in olive oil (5–20 mg/100 g) [68]. HT has been thoroughly studied in terms of its aging, neurodegenerative diseases, metabolic disorders, and CVD [68]. The EFSA [69] claimed that 5 mg of HT and its derivatives (such as tyrosol and oleuropein) should be consumed daily to reduce oxidative stress and protect LDL from oxidative damage. Although the meta-analysis conducted by Pastor et al. [70] found that HT had no effect on MetS, HT supplementation could affect the antioxidant capacity components associated with MetS. Some clinical trials involving patients with metabolic disturbances have shown that HT supplementation (5–9.7 mg/day) could improve insulin sensitivity, blood pressure (BP), and lipid profile [71,72,73]. HT administration (9.3 mg/g) for 8 weeks reduced LDL, oxidized LDL, total cholesterol, triglycerides (TG), and apolipoprotein B (ApoB) in MetS patients [71]. In vitro studies confirmed that HT can regulate lipid metabolism by activating AMP-activated protein kinase (AMPK) and decreasing the enzymatic activity of acetyl-CoA carboxylase (ACC), diacylglycerol acyltransferase (DGAT), and 3-hydroxy-3-methyl-glutaryl-CoA reductase (HMGCR) [74]. In 3T3-L1 adipocytes, HT upregulates the expression of carnitine palmitoyltransferase 1 (*CPT-1*) and peroxisome proliferator-activated receptor *α* and *γ* genes (*PPAR-α* and *PPAR-γ*). Moreover, it effectively stimulates mitochondrial biogenesis and mtDNA quantity, affects mitochondrial function, and lowers free fatty acid (FFA) levels in adipocytes. This action could explain the hypolipemic and insulin-sensitizing effect of HT [75].

To the best of our knowledge, no research has been conducted on the potential of table olives as a food that could alleviate MetS. However, due to its chemical composition, which is determined by both the biologically active compounds contained in the raw fruits and those formed as a result of the fermentation process, table olives could be considered a potential superfood for improving metabolic disturbances. Further investigation is needed to determine the impact of table olives consumption on MetS components.

### 4.2. Capers

The caper (*Capparis spinosa*) is known as a medicinal plant in Iranian Traditional Medicine. Pickled caper buds and berries are edible products and are produced mainly in Mediterranean countries. Although fresh caper fruits used to be consumed by elderly Iranian and Bahrainis, the consumption of raw caper berries is not popular due to their bitter taste caused by the presence of the glucosinolate glucocaparin. To eliminate bitterness, caper buds and berries undergo spontaneous lactic acid fermentation in brine or a mixture of brine and vinegar. Fermented caper buds are appreciated for their distinctive sensory attributes, including their characteristic flavor and texture [31,76,77,78].

#### 4.2.1. Nutrients and Phytocompounds

The main phytocompounds in fermented caper buds are flavonols, mainly kaempferol-3-*O*-rutinoside (409.85 mg/100 g dry weight (DW)) and quercetin-3-*O*-rutinoside (rutin) (316.35 mg/100 g DW) (Table 1). These flavonols also occur in caper berries, but their content is much lower (1.66 and 12.12 mg/100 g, respectively) [31]. Brine-fermented caper fruits also contain FA (mainly linoleic, oleic, and palmitic) [79] and phenolic acids (including ferulic acid, gallic acid, and protocatechuic acids) [31,32,79]. Glucosinolates present in raw berries are fully degraded during fermentation [76]. Caper fruits are also a good source of some minerals, including potassium (994.25 mg/100 g fruits), calcium (327.72 mg/100 g), and magnesium (109.32 mg/100 g) [79].

#### 4.2.2. Microbiota

The bacterial community of caper fruits includes LAB (*Lactobacillus*, *Pediococcus*) and *Enterococcus faecium*. The predominant species of bacteria is *L. plantarum* [80]. The only yeast species identified in caper pickles is *Aureobasidium pullulans* (Table 1) [33]. There were attempts to obtain pickled caper berries with a starter strain (*L. pentosus* OM13). The use of a starter strain seems promising because the product is characterized by a low bitterness and high hardness [33]. To the best of our knowledge, there is a lack of precise information about microbiota in fermented caper buds. Ózcan et al. [81] reported that the LAB group occurs in fermented bud brine during 10–20 days of fermentation. The quantity of LAB depends on the bud size and is higher in small ones [81].

#### 4.2.3. The Relevance of Capers in the Context of MetS

Although fermented capers, compared to raw ones, have a higher TPC and total flavonoid content, stronger antioxidant properties, and are a source of probiotic bacteria, there are few studies on their health-promoting properties [76,80]. Two clinical studies confirmed that pickled caper fruit consumption (12 weeks) could exert anti-diabetic and hypolipidemic effects in overweight and obese patients with non-alcoholic fatty liver disease (Table 2) [77,82]. Pickled caper fruits may also enhance the hypolipemic effect of statins [83]. To the best of our knowledge, no one has so far studied the metabolic properties of caper bud pickles.

## 5. Fermented Vegetables

The chemical composition, microbiota, and health-promoting properties of selected fermented vegetable products are summarized below.

### 5.1. Kimchi

Kimchi is a traditional Korean fermented dish prepared from Chinese cabbage, leeks, radishes, chives, and condiments such as ginger, garlic, red pepper powder, and salted fermented seafood (aekjeot) or fish sauce (jeotgal). Kimchi has been an integral part of Korean food culture for thousands of years, and in 2013, the communal activity of making kimchi was inscribed on UNESCO’s list of Intangible Cultural Heritage of Humanity. Kimchi has gained popularity worldwide for its health benefits. Due to the fermentation process and the selection of spices, it is characterized by a unique spicy, sour, sweet, and carbonated taste. Fermentation is conducted at low temperatures and in anaerobic environments [16,34,35,123,124].

#### 5.1.1. Nutrients and Phytocompounds

Kimchi contains 17.9 g of protein, 2.3 g of lipids, 31.3 g of dietary fiber (both soluble and insoluble), microelements such as potassium (44.3 mg/100 g), calcium (1.5 mg/100 g), magnesium (1.7 mg/100 g), zinc (33.9 mg/100 g), and vitamins [125]. The chemical composition is determined by the range of condiments used for product preparation. The addition of red pepper is a key source of vitamin C, carotene, and capsaicin, while the addition of ginger provides anti-inflammatory phytocompounds, gingerol, and shogaol [126]. The basal kimchi ingredient, cabbage, is a source of phytoestrogens. Among them is *β*-sitosterol, which competes for absorption with cholesterol. Leeks, onions, garlic, and spring onions are sources of sulfur compounds. Also, kimchi cabbage contains sulfur compounds, including sulforaphane, allyl isothiocyanate, benzyl isothiocyanate, and phenyl isothiocyanate [36,126]. However, during fermentation, the amount of isothiocyanates, especially sulforaphane, decreases [127]. In fermented products, there also occur volatile sulfur compounds (allyl methyl disulfide, diallyl tetrasulfide, 4-ethyl-5-methylthiazole, allyl methyl trisulfide, 3-vinyl-[4H]-1,2-dithiin) and 2-phenylethyl isothiocyanate [128]. The relevant biologically active compound is 3-(40-hydroxyl-30,50-dimethoxyphenyl) propionic acid (HDMPPA). A study on an animal model confirmed that supplementation with HDMPPA has a beneficial impact on lipid profile (decrease of TG and LDL) and an anti-atherogenic effect [36]. The chemical composition of kimchi is presented in Table 1.

#### 5.1.2. Microbiota

Kimchi contains a complex microbial ecosystem, including LAB (*Lactobacillus*, *Lactococcus*, *Leuconostoc*, *Pediococcus*, *Tetragenococcus*, and *Weissella*), yeasts (*Candida*, *Kluyveromyces*, *Lodderomyces*, *Pichia*, *Saccharomyces*, and *Trichosporon*), and archaea (Table 1) [16,34,35,124]. The composition of the microbial community and its metabolic profile are determined by the microbial niches in the raw ingredients [17], salt concentration [129], and the duration of fermentation [16]. The LAB present in kimchi originates mainly from vegetables, with the exception of *Tetragenococcus*, which comes from jeotgal [35]. Yeasts that occur during the late stage of fermentation are responsible for an undesirable white film on the surface of the product [16]. LAB originating from kimchi is responsible for the production of metabolites such as *γ*-aminobutyric acid (GABA), bacteriocins, ornithine, exopolysaccharides, etc. The mentioned metabolites enable them to survive in an acidic environment. Bacteriocins are proteins or peptides synthesized in bacterial ribosomes. Due to antibacterial properties, bacteriocins could be applied as natural food preservatives. They originated from kimchi LAB strains, and their stability has been summarized by Lee et al. [124]. One of the most significant metabolites is GABA, produced primarily by L. brevis [124]. Due to the multidirectional health-promoting properties of GABA, there have been attempts to increase its content in kimchi. The most effective manner seems to be inoculation by GABA-producing LAB (for instance, *Lactobacillus zymae* (currently *Levilactobacillus zymae*) GU240) and the addition of the direct GABA precursor monosodium glutamate [124]. Other important bacterial metabolites that occur in kimchi are lactic acid and acetic acid, which are responsible for the flavor and aroma of kimchi [128].

#### 5.1.3. The Relevance of Kimchi in the Context of MetS

Kimchi consumption or supplementation with kimchi-derived probiotic strains is associated with various health-promoting effects, proven in both in vitro and animal and clinical studies (Table 2). Kimchi or kimchi LAB strains have antioxidant [36], anti-inflammatory [16,85,130], anti-obesity [16,84,85,86,130,131,132], anti-diabetic [36,84,85,86,130,133], hypotensive [86], and hypolipidemic effects [16,36,84,85,130]. In the Korean Genome and Epidemiology Study (with 10-year follow-up), frequent consumption of kimchi was correlated with a lower risk of MetS in women, while in men, kimchi consumption was not associated with MetS [134].

An et al. [86] compared the anti-diabetic, hypotensive, and anti-obesity effects of kimchi before and after fermentation. Both types of kimchi, consumed daily for 8 weeks, improved body weight, body mass index (BMI), and waist circumference (WC), but only fermented kimchi improved BP. Insulin sensitivity, measured by the Matsuda Index and Homeostatic Model Assessment for Insulin Resistance (HOMA-IR), was improved by both types of kimchi, but the Quantitative Insulin Sensitivity Check Index (QUICKI) and Disposition Index (DI) were improved only after consumption of the fermented product. It is possible to improve the health-promoting effect and production of kimchi by adding a starter culture. Consumption of fermented kimchi with *Weissella koreensis* OK1-6 was associated with lower serum leptin and insulin concentrations and a better lipid profile and metabolism in mice maintained on a high-fat diet. The effect after consumption of kimchi manufactured without a starter was also significant but weaker [85]. Furthermore, kimchi fermented with *W. koreensis* OK1-6 contains a higher amount of ornithine than spontaneously fermented kimchi (117.06 vs. 21.43 mg/100 g fresh weight). It is suggested that in addition to probiotic bacteria, ornithine could be responsible for the anti-obesity effect of kimchi [85]. Moreover, kimchi fermented with starter *W. koreensis* OK1-6 [85] and the probiotic strain isolated from kimchi *L. brevis* OPK-3 [130] modify lipid metabolism in the liver by downregulating genes related to lipid metabolism (liver X receptor alpha gene (*LXRα*), sterol regulatory element-binding protein 1c gene (*SREBP-1c*), *ACC*, sterol regulatory element-binding protein 2 gene (*SREBP-2*), *PPAR-γ*, fatty acid synthase gene (*FAS*), low-density lipoprotein receptor gene (*Ldlr*), 1-acyl-sn-glycerol-3-phosphate acyltransferase 5 gene (*Agpat5*)) and upregulating genes related to FA oxidation and lipid catabolism (*Ppar-α*, acyl-CoA oxidase gene (*Acox2*), *Cpt-1a*). Probiotic strains from fermented products that are beneficial in terms of metabolic health are summarized in Table S1.

### 5.2. Fermented Cabbage and Sauerkraut

The term “sauerkraut” comes from German and means “sour cabbage”. Fermented cabbage probably originated in China, where cabbage was fermented with the addition of wine over 2000 years ago. In Europe, fermented cabbage was first produced 1000 years later, and in its production, wine was replaced by salt [135]. It is prepared from *Brassica oleracea* var. *capitata* [37,38], while in China, it is prepared from the Chinese cabbage (*Brassica pekinesis*) [136,137]. For sauerkraut preparation, cabbage is shredded into strips and compressed in jars or tanks with the addition of salt in the amount of 2–2.5%. Tight compression of cabbage is critical to obtaining the anaerobic conditions that promote LAB growth [38,135]. European sauerkraut is fermented in cabbage juice, which occurs after compressing cabbage, whereas Chinese sauerkraut is fermented in water [136]. The ready-to-eat product should be characterized by a sour taste and high hardness and crunchiness [38]. Pickled cabbage is also a traditional product of Turkey, where large pieces of cabbage are fermented in brine containing 6–8% salt [138].

#### 5.2.1. Nutrients and Phytocompounds

Sauerkraut contains 4.28 g of carbohydrates, 0.14 g of fat, and 2.9 g of fiber. It is also a source of minerals, including sodium, potassium, and calcium, as well as vitamins K and C [135,138]. Historically, sauerkraut was an important source of vitamin C, which protected sailors from scurvy [135].

Fermented cabbage contains organic acids (lactic acid, malic acid), short-chain fatty acids (SCFAs), aldehydes (acetylaldehyde), esters (ethyl acetate, ethyl lactate), amino acids, alcohols, exopolysaccharides, biogenic amines, and other compounds [37,38,39]. Sauerkraut’s flavor is associated mainly with the presence of amino acids and organic acids [40]. In sauerkraut brine, free amino acids, including aromatic amino acids, significantly increase during fermentation [37]. Among SCFAs, acetic acid and butyric acid considerably increase during fermentation. The abundance of lactic acid, acetic acid, and some amino acids (Phe, Tyr) in brine is correlated with the presence of *Lactiplantibacillus* [37]. Spontaneous fermentation of sauerkraut was positively correlated with tartaric acid, malic acid, and lactic acid [40]. GABA was detected only in the early stage of fermentation. GABA content is positively correlated with the abundance of *Pantoea*, a bacterium that naturally colonizes *Brassica* leaves and metabolizes isothiocyanates during fermentation [37]. Glucosinolates in raw cabbage also undergo enzymatic degradation by the endogenous enzyme myrosinase. In fermented sauerkraut, only glucosinolate metabolites (including isothiocyanates and cyanides) have been identified. The predominant metabolite of glucobrassicin, one of the main glucosinolates in cabbage, is ascorbigen, a product of the reaction of indole-3-carbinol with vitamin C [41].

Methanol is the most abundant alcohol in sauerkraut. It is probably formed as a product of the enzymatic degradation of pectins contained in cabbage. It influences the sensory features of sauerkraut because it is a substrate that forms methyl esters. Among the terpenes, the most prevalent observed were geranyl acetone and *β*-damascenone [38]. Additionally, some biogenic amines, including tyramine and, in smaller amounts, putrescine, histamine, tryptamine, and cadaverine, were identified in sauerkraut (Table 1). The amount of biogenic amines is positively correlated with the presence of yeasts [38].

#### 5.2.2. Microbiota

The fermentation process can be divided into three stages. The first phase (a ‘turbulent fermentation’) lasts 2–3 days [42]. The primary stage of fermentation is carried out by bacteria occurring in raw cabbage, including *Pantoea*, *Serratia*, *Pseudomonas*, *Enterobacter*, *Pectobacterium*, *Lelliottia*, *Acinetobacter*, and *Buttiauxella* [37]. During the first phase, the amount of oxygen and pH decrease, and extensive gas production occurs. Subsequently, a higher abundance of bacteria from the family *Lactobacillaceae* is observed, including species such as *Lactiplantibacillus*, *Levilactobacillus*, *Secundilactobacillus*, *Pediococcus*, *Lentilactobacillus*, *Leuconostoc*, and *Paucilactobacillus* [37]. The final phase (maturation) lasts up to several months and is crucial for determining the sensory attributes of the final product [135]. In the final product, *L. plantarum* is the predominant bacterium [37,39]. Aside from LAB, yeasts are also responsible for the unique taste, texture, and preservation of fermented cabbage. The yeasts found in sauerkraut include *Pichia*, *Wickerhamomyces*, *Rhodotorula*, *Debaryomyces*, and *Clavispora* [139]. Some yeast species, for instance, *Debaryomyces hansenii* and *Cryptococcus macerans*, may contribute to the shortening of shelf life and symptoms of spoilage [42]. There have also been attempts to use yeast as starter strains for sauerkraut fermentation [40,139]. The detailed characteristics of the microbial community of fermented cabbage are shown in Table 1.

#### 5.2.3. The Relevance of Sauerkraut and Fermented Cabbage in the Context of MetS

Sauerkraut is a product with antioxidant [136,138] and anti-inflammatory properties [37]. Antioxidant properties can be attributed to the presence of antioxidant exopolysaccharides produced by *Lactobacillus paracasei* (currently *Lacticaseibacillus paracasei*), present in Indian pickled cabbage [140].

To the best of our knowledge, there are no in vivo studies on the effect of sauerkraut consumption on lipid and carbohydrate metabolism. However, there is research on the effects of particular *L. plantarum* strains isolated from fermented cabbage on components of the MetS. *L. plantarum* S4-1, isolated from Chinese sauerkraut, could be applied as a probiotic. *L. plantarum* S4-1 was able to assimilate cholesterol in vitro (in a medium), and milk fermented with this strain reduced serum cholesterol in mice [141]. *L. plantarum* S9, isolated from sauerkraut, is a probiotic strain with possible application in the prevention of MetS. Oral administration of *L. plantarum* S9 in MetS rodents resulted in an improvement in body weight, inflammation, insulin sensitivity, lipid profile, and accumulation of fat in the liver. The anti-inflammatory action was via the TLR4/NF-κB pathway. Probiotic supplementation was associated with a decreased expression of toll-like receptor 4 (TLR4), which is a receptor for bacterial lipopolysaccharide, inhibition of nuclear factor-κB (NF-κB) pathway activation, and decreased expression of inflammatory factors [142]. Furthermore, *L. plantarum* H31 may be considered a probiotic strain in the prevention and alleviation of diabetes. Exopolysaccharide from *L. plantarum* H31 inhibits *α*-amylase and promotes glucose uptake in hepatic cells via enhancement of the glucose transporter-4 gene (*GLUT-4*), protein kinase B gene (*Akt-2*), and *AMPK* expression [143].

## 6. Fermented Legumes

The chemical composition, microbiota, and health-promoting properties of fermented legumes are discussed below.

### 6.1. Fermented Soybean Products

Soybean (*Glycine max* (L.) Merr.) is a legume indigenous to Asian countries. Recently, soybeans have become one of the most popular oilseed plants and the cheapest source of plant-derived protein worldwide [144,145]. Raw seeds contain about 40% protein, 35% carbohydrates, 8–24% lipids, and dietary fiber [145]. A crucial group of biologically active compounds found in soybeans is isoflavones, followed by vitamins, saponins, flavonoids, and phenolic acids [144,146]. Soybean seeds contain 12 isoflavone isomers, such as aglycones (daidzein, genistein, and glycitein) and their corresponding *β*-glucosides, acetyl-glucosides, and malonylglucosides [44]. The biologically active compounds also include anti-nutritional compounds such as trypsin inhibitors and phytic acid [144,146]. Soybean is known as a functional food with cardioprotective properties. The meta-analysis conducted by Mohammadifard et al. [147] concluded that soy consumption has a statistically significant and favorable impact on lipid profile and glycemic parameters (fasting blood glucose (FBG), insulin level, and HOMA-IR) in patients with MetS [147].

In Asia, soybeans provide the basis for the production of various fermented products, for instance, natto, tempeh, kinema, douchi, cheonggukjang (CGJ), doenjang, miso, and fermented soy milk. The microbial communities responsible for soy fermentation can be bacteria (mainly *Bacillus*), mold (*Rhizopus*), or both [146]. Fermentation enhances the digestibility and nutritional value of soybeans, including an increase in the amount of free amino acids, vitamins, and GABA and the degradation of anti-nutritional compounds [45,146,148,149]. Furthermore, isoflavone glycosides, the predominant form of isoflavones in raw soybeans, are hydrolyzed in the presence of microbial *β*-glucosidases to more active aglycones with higher bioavailability [44,105].

#### 6.1.1. Tempeh

Tempeh (or tempe) is a compact, cake-like product prepared from boiled soybeans by fermentation with *Rhizopus*. The product should have a firm texture, a nutty, mushroom-like flavor, and a white color, which is given by *Rhizopus* mycelium. Tempeh is a traditional soybean product that originated from Indonesia. It provides a good alternative to animal protein and is a staple protein source for Indonesians [150,151,152]. Production of tempeh includes steps such as soaking, dehulling, boiling, draining, and inoculating with spores of *Rhizopus* spp. The product is packed in leaves or perforated bags and incubated in semi-aerobic conditions. Tempeh can be prepared in different ways, including boiling, steaming, frying, or grilling. As a raw material for tempeh production, apart from soybeans, other legumes (chickpea, black gram, peas, and beans), grains (such as wheat, oats, and barley), or nuts may be used [150].

##### Nutrients and Phytocompounds

The unique nutritional value and chemical composition of tempeh are closely related to the enzymatic activity of the mold. *Rhizopus* produces enzymes, including proteases, lipases, and amylases, which are responsible for the degradation of complex compounds contained in soybeans [150,151]. Processing raw soybeans into tempeh results in some favorable nutritional changes, such as the degradation of starch and flatulence-causing oligosaccharides (raffinose and stachyose), and increasing protein quality and amounts of B_2_ and B_3_ vitamins [153,154,155]. *Rhizopus* also produces phytases that degrade phytic acid, resulting in the increased bioavailability of minerals such as iron, zinc, and magnesium, which are bound to phytate [148]. During fermentation, soy protein (albumin, globulin, and alkaline soluble proteins) degrades to amino acids and biologically active peptides. Amino acids, present in the largest amounts after 48 h of fermentation, are alanine, glutamic acid, and glycine. Fermentation also increases the number of essential amino acids, except for methionine. The amount of leucine is more than doubled, while the amount of phenylalanine increases more than four-fold [45,149]. Enhancement of lipase activity during fermentation results in an increase in FFA [156]. Additionally, during fermentation, the amount of GABA gradually increases (21.4 mg/100 g soybean/24 h) [45].

In raw soybean seeds, more than 99% of isoflavones occur, mainly as glucosides. Due to the high activity of *β*-glucosidase, produced by *R. oligosporus* during tempeh fermentation, the aglycone content increases over 20 times. The isoflavones contained in tempeh are daidzein, glycitein, and genistein, and their *β*-glucosides daidzin, glycitin, and genistin (Table 1). Daidzin and daidzein occur after 3 days of fermentation in high amounts (225.2 and 374.8 μmol/100 g), while glycitin and glycitein are present in smaller amounts (195.9 and 33.1 μmol/100 g) [44].

##### Microbiota

Currently, inoculation of soybeans by mold—*Rhizopus* spp.—is used in tempeh production. Traditionally, soybeans were wrapped into leaves, which led to accidental inoculation by the *Rhizopus* present on the leaf’s surface [150]. Among *Rhizopus* species, the most common is *R. microsporus*, while the amount of other species has declined due to the commercialization of tempeh production [46]. Among bacteria, the predominant phylum was found to be Firmicutes, and the predominant order was found to be *Lactobacillales*, including *Lactobacillus agilis* (currently *Ligilactobacillus agilis*), *Lactobacillus fermentum* (currently *Limosilactobacillus fermentum*), *Lactobacillus mucosae* (currently *Limosilactobacillus mucosae*), and *Lactobacillus delbrucki* [47]. The microbial community of tempeh also includes other bacteria (*Clostridium*), molds (*Mucor* and *Rhizomucor*), and Zygomycetes fungi (*Absidia*) (Table 1) [48,49]. Different modifications of the inoculum can be applied for special purposes. The addition of *S. cerevisiae* to the inoculum could improve tempeh’s health-promoting effect by enhancing *β*-glucan’s content and antibacterial activity against *Escherichia coli* [157], while *Propionibacterium* could be added to increase the amount of vitamin B_12_ [50].

##### The Relevance of Tempeh in the Context of MetS

Although tempeh is a valuable source of bioactive compounds and displays significant health-promoting properties, in Indonesia, it is still considered a “low-class” protein that is available to people who cannot afford meat [150]. In vivo and human studies confirmed anti-obesity [107,109], hypoglycemic [101,102,103], and hypolipidemic [100,102,106,108] effects of tempeh consumption (Table 2). Animal studies have demonstrated that the administration of tempeh can improve parameters connected with diabetes, including FBG, HbA1c, HOMA-IR, and oral glucose tolerance tests (OGTT) [98]. A decrease in HbA1c was also observed in diabetic patients after supplementation with freeze-dried tempeh [104]. In the diabetic rat model, tempeh administration improves the lipid profile by decreasing the concentration of total cholesterol (TC), LDL, and TG and increasing HDL levels [98]. The hypolipemic and hypoglycemic properties of tempeh seem to occur due to the probiotic properties of LAB, the high dietary fiber content, SCFAs, soy isoflavones, and soy protein [98,100,106,107,152]. Moreover, some bioactive peptides formed during fermentation can act as *α*-glucosidase, dipeptidyl peptidase 4 (DPP IV), and angiotensin-converting enzyme (ACE) inhibitors [149,158]. The chemical composition of tempeh, including free amino acids, peptides, and isoflavone aglycones, also provides antioxidant properties and decreases the amount of malondialdehyde, which is a product of lipid peroxidation [100,159,160]. Tempeh produced from germinated soybeans possesses stronger antioxidant activity and a higher protein content than tempeh produced from non-germinated soybeans. On the other hand, tempeh produced from non-germinated soybeans contains more isoflavones (116.02 vs. 80.13/ 100 g tempeh flour) and exhibits stronger *α*-amylase and *α*-glucosidase inhibition [161]. Furthermore, tempeh is a good source of resistant starch, including arabinoxylan (AX), which prevents TG liver accumulation, decreases FAS activity, blood glucose, and insulin levels, and improves the postprandial glycemic response. AX intake is also connected with an increase in the amount and activity of butyrate-producing gut *Roseburia* spp., which is decreased in diabetic subjects [152,162].

#### 6.1.2. Natto

Natto is a fermented soybean product characterized by a sticky and slippery texture, nutty flavor, and sour aroma [163]. It is believed that natto originated either from the Yunnan province of China or from the Tohoku region of Japan, where it was invented by accident when boiled, spoiled soybeans were eaten and thought to be tasty. Over the years, natto gained popularity mainly in Japan. In the 1980s, natto appeared in supermarkets, and its consumption rose rapidly. Production includes steps such as soaking, steaming under pressure, inoculation, fermentation (40 °C, 16–18 h), and maturation. Natto is usually mixed with egg and condiments and served with rice for breakfast [163,164].

##### Nutrients and Phytocompounds

Natto contains 19 g of protein, 11 g of fat, and 5.4 g of dietary fiber per 100 g. It also provides large amounts of iron (8.6 mg/100 g), calcium (217 mg/100 g), and potassium (729 mg/100 g) [165]. The quantity of vitamin K_2_ considerably rises during fermentation, and its final content is 124 times higher than in unfermented soybean (880 mg/100 g) [163]. The fermentation process also affects the quality and quantity of amino acids. During fermentation, the amount of proline increases, contents of threonine, aspartic acid, serine, and valine are stable, while the amount of 11 other amino acids decreases dramatically [43]. Among the SCFAs, branched SCFAs (isobutyric and isovaleric acids) were detected, while their straight-chain isoforms (butyric and valeric) were absent [43]. Isoflavone aglycones are the major group of biologically active compounds in natto [43], followed by biogenic amines (spermine, spermidine, and tyramine) [166], exopolysaccharides (levan) [167], as well as volatile compounds, ketones, and pyrazines (Table 1) [43].

##### Microbiota

Before the isolation of a starter strain, rice straw, which is a natural habitat for *B. subtilis* spores, was used to initiate fermentation. Now, natto production uses a fermentation process with a starter culture of *B. subtilis* var. natto [168]. *B. natto* produces two crucial metabolites: *γ*-polyglutamic acid (*γ*-PGA) and nattokinase (NK) [169,170].

*γ*-PGA is a biopolymer consisting of glutamic monomers linked by peptide bonds between α-amino and *γ*-carboxylic groups [170]. It is responsible for the characteristic sticky consistency of natto [163]. Administration of *γ*-PGA in diabetic mice resulted in improved lipid metabolism, insulin resistance, HOMA-IR, reduced FBG, and enhanced expression of genes associated with the insulin pathway: INSR, Akt, IRS-1, and PI3K [171]. In the diet-induced obesity mouse model, *γ*-PGA supplementation reduced adipocyte size and improved the lipid profile (TG, LDL, and HDL levels), as well as parameters connected with glucose metabolism (HOMA-IR, insulin, and glucose levels). Supplementation was associated with a lower BW and food intake. *γ*-PGA influences AMPK, PPAR-*γ*, CPT-1, and SREBP-1 mRNA expression in the liver and adiponectin, leptin, GLUT-4, and PPAR-*γ* expression in white adipose tissue [172]. Moreover, *γ*-PGA consumption was associated with a reduction in visceral fat accumulation [173], while coadministration with levan (exopolysaccharide present in natto) also decreased epididymal fat, fat cell size, and leptin levels [169].

NK is an alkaline serine protease that exhibits robust antithrombotic and fibrinolytic activity [169]. NK is an ACE inhibitor [174,175]. Its supplementation reveals hypotensive effects both in Korean and in North American populations [176,177]. Supplementation with NK for 8 weeks decreased both systolic and diastolic BP [176,177] and reduced angiotensin (AGT) activity [177]. The hypotensive effect was more pronounced in the male population [176]. Following NK supplementation, a slight improvement in the lipid profile in patients with primary hypercholesterolemia was observed, but the effect was not statistically significant [178].

##### The Relevance of Natto in the Context of MetS

Natto could be considered as a food suitable for patients with MetS due to its antioxidative [110,112,114], anti-glycative [114], anti-obesity [112,113,179], anti-inflammatory [169,180], hypoglycemic [113,181,182,183,184,185], hypotensive [186], and hypolipidemic [111,112,113,114,180,185] properties (Table 2). It is worth noting that natto has a greater impact on glucose and lipid metabolism as well as on BP than unfermented soybeans [112,113,186].

Probably, *γ*-PGA, polysaccharides, and dipeptides are responsible for the hypoglycemic effect of natto [113,181,182,183,184] from *γ*-PGA-rich natto, which lowered the glucose incremental area under the curve and suppressed high postprandial glucose in the early stage after a meal. In addition, a meal consisting of rice and PGA-rich natto suppressed insulin secretion more than rice with low-PGA-content natto [182]. In addition, natto-derived polysaccharides also suppressed hyperglycemia and inhibited glucose uptake in vitro [183], while two peptides isolated from natto water extracts (Lys-Leu and Leu-Arg) were resistant to digestion and acted as DPP IV inhibitors [184].

The hypotensive effect of natto is probably due to ACE inhibitory properties, revealed by both NK [174,175] and dipeptides [186]. Seventy-two natto dipeptides have significant ACE inhibitory properties, and among them, the strongest enzyme inhibition was reported for Glu-Norval, Glu-Lys, and Thr-Arg [186].

Changes in lipid metabolism, including the inhibition of FA synthesis in the liver and FA catabolism, are responsible for natto’s anti-obesity effect. Natto suppresses FA synthesis in the liver by decreasing the expression of Fas, malic enzyme (Me), glucose-6-phosphate dehydrogenase X-linked (G6pdx) genes, and the transcription factor for these genes (Srebp-1c). Natto promotes FA catabolism in the liver by increasing the expression of the Cpt1 gene, which promotes FA *β*-oxidation, a consequence of a high-fat diet (HFD). Natto did not affect the transcription of other genes connected with *β*-oxidation (acyl-CoA oxidase gene (Aco), Cpt2, and Ppar-α) [112,113]. Moreover, the anti-obesity effect and improved lipid metabolism after natto consumption may occur as a consequence of increased expression of cholesterol 7 alpha-hydroxylase, decreased expression of sterol-regulatory element-binding protein and HMGCR, and suppressed activity of LXR target genes [179].

*B. natto* may be considered as a probiotic addressed to patients with metabolic disturbances. Supplementation with *B. natto* alleviates diet-induced obesity, visceral fat accumulation, and low-grade inflammation. Lower lipid accumulation and the hypolipidemic effect after *B. natto* supplementation are probably due to an increased expression of Ppar-α and decreased expression of Srebp-1c, while the anti-obesity effects are due to an increase in p-PI3K/PI3K, p-AKT/AKT, and IRS-1 protein expression. Although *B. natto* supplementation resulted in improvements in all parameters of the lipid profile, the most significant difference was reported for HDL, which increased by 60% after supplementation with *B. natto* at a dose of 1× 1010 CFU/mL (compared to the HFD group) [180,185].

Natto causes favorable changes in gut microbiota. Its consumption improves the Bacteroidetes/Firmicutes ratio, which is lower in subjects on HFD. Its consumption is associated with a decrease in *Parabacteroidetes*, connected with obesity and type 2 diabetes, and an increase in *Allobaculum*, which is associated with improvements in obesity-related parameters [112,113]. Moreover, *B. natto* supplementation promotes the growth of *Verrucomicrobias*. The main strain of *Verrucomicrobias*, *A. muciniphila*, is a probiotic strain adversely associated with obesity, low-grade inflammation, and diabetes [180].

#### 6.1.3. Cheonggukjang

CGJ is a traditional Korean product manufactured from boiled soybeans. CGJ fermentation is induced by rice straw (a natural *B. subtilis* habitat) or by a commercially available starter culture (*B. subtilis*). The production includes steps such as soaking, cooking (121 °C, 0.5 h), cooling, inoculation, fermentation (40 °C, 48–72 h), and maturation. In contrast to fermented soybean products and traditional Korean fermented foods, CGJ does not contain salt. CGJ is often consumed as a gravy, served with boiled rice [51,187]. The main obstacle that limits CGJ consumption is its unpleasant smell while cooking. To improve its sensory qualities, there have been attempts to prepare CGJ with the addition of green tea, kiwi, radish, or red ginseng [188].

##### Nutrients and Phytocompounds

CGJ contains 20.9 g of protein and 18.4 g of fat per 100 g [52]. The most abundant amino acids are leucine and glutamic acid, followed by tyrosine, proline, and alanine [53]. As well as the above-mentioned products, CGJ is a significant source of physiologically active compounds, including dietary fiber, isoflavone aglycones, unsaturated FA, saponins, biogenic amines, GABA, *γ*-PGA, oligosaccharides, and enzymes (protease, cellulase, amylase) [51,52,189,190]. Characteristic compounds absent in the unfermented soybean include hydrocarbons (benzene, dichloromethane, 2,2,6-trimethyl decane), ketones (1,3-diphenyl-2-propanone, 2,3-butanedione, acetone), alcohols (2,3-butanediol), aldehydes, acids (lactic acid), esters, pyrazines, and aromatic compounds [53,54,191]. A compound that deserves attention for its beneficial health-promoting properties appears to be 1,3-diphenyl-2-propanone, which is a PPAR*α*/*γ* dual agonist [54]. The detailed characteristics of the chemical composition of CGJ are shown in Table 1.

Similar to the previously described soybean fermented foods, CGJ contains biogenic amines (mostly tyramine, followed by *β*-phenylethylamine and putrescine [189]). The level of some biogenic amines in CGJ exceeds the safety level [189]. To reduce the content of biogenic amines, selected strains (e.g., *B. licheniformis*) [192] or some food additives (tartaric acid, sodium benzoate, potassium sorbate) could be applied [193].

##### Microbiota

The microbial community of naturally fermented Korean CGJ mainly consists of bacteria (95.83%), followed by viruses (2.26%), unclassified species (1.84%), eukaryotes, and archaea. The bacterial community of CGJ mainly consists of *Bacillus* (91.55%), followed by *Brevibacillus*, *Acinetobacter*, *Carnobacterium*, *Paenibacillus*, *Cronobacter Enterococcus*, *Enterobacter*, *Terriglobus*, *Psychrobacter*, *and Virgibacillus* (Table 1). In CGJ, 150 species of *Bacillus* have been identified. According to Tamang et al. [187], the most abundant *Bacillus* species is *B. thermoamylovorans*, while according to Jin et al. [193], the most prevalent species is *B. piscis* or *B. licheniformis* (depending on the method used). CGJ fermentation may be conducted with starter cultures, mainly *Bacillus* spp., but also *Leuconostoc* or *Enterococcus* [51].

##### The Relevance of CGJ in the Context of MetS

Consumption of tempeh or administration of CGJ as a fresh food or freeze-dried powder enhances the antioxidative status [96,194], mitigates inflammation [93,94], and improves the metabolic parameters (Table 2) [95]. CGJ exhibits an anti-diabetic effect [99], including an improvement in FBG levels [87,90,195], an insulin-sensitizing effect [196,197], and protection from *β*-cell apoptosis [92]. The molecular mechanism underlying the regulation of glucose metabolism and utilization is an enhanced expression of *PPAR-γ* and *GLUT4* [195,196]. Water extracts from traditionally fermented CGJ (which contain mostly peptides) have stronger insulin-sensitizing properties (via increasing expression of *PPAR-γ*) than unfermented soybeans. The CGJ methanol extract (rich in isoflavone aglycones) protects *β*-cell viability and promotes insulin secretion in insulinoma cells more strongly than unfermented soybeans [197]. *β*-cell survival after CGJ consumption is also supported by stimulating the insulin/IGF-1 signaling cascade [195]. CGJ has an impact on blood glucose by suppressing the expression of *PEPCK*, a gene involved in gluconeogenesis, which is upregulated by HFD [92].

CGJ administration was associated with decreased body weight and improvement in levels of appetite-regulating hormones (both leptin and adiponectin) [87,88,92]. The anti-obesity effect of soybeans fermented by *B. subtilis* MORI, isolated from CGJ, was demonstrated by the suppression of preadipocyte differentiation, by decreased CCAAT element-binding protein *α* (*CEBP/α*) expression, and by increased ACC phosphorylation, although this action was reported only in vitro on 3T3-L1 preadipocytes [190,196]. Additionally, saponins contained in CGJ, mostly soyasapogenol B, enhance AMPK activation, which leads to FA synthase being switched off by phosphorylation and, consequently, inactivation of ACC [74,190]. The anti-obesity action is also associated with *γ*-PGA content and changes in isoflavones during fermentation. CGJ, in a traditional manner, reduced body weight and epididymal fat less effectively than a product fermented by a *γ*-PGA-producing strain [87].

The hypolipidemic effect of CGJ has been confirmed in both animal and human studies [87,89,90,91,94,96,198]. CGJ improved lipid metabolism by decreasing the expression of the transcription factors *SREBP-1c* and *CEBP/α*, both in hepatic and adipose tissue. Suppression of *SREBP-1* inhibits hepatic lipogenesis and, in consequence, lipid accumulation, while lowering *CEBP/α* expression results in the reduced expression of adipogenic effector genes [94]. Moreover, CGJ supplementation could regulate lipid metabolism by increasing the liver expression of *CPT-1* and *ACO*—genes connected to the regulation of FA *β*-oxidation [89]. Despite numerous studies confirming the beneficial effect of CGJ on metabolic parameters, Han et al. [97] reported no effect of CGJ consumption on inflammation, lipid profile, body weight, and glucose tolerance.

#### 6.1.4. Kochujang

Kochujang is a Korean red pepper paste used as a sauce for meat, a dressing for vegetables, or a seasoning for soup and stew [115]. Traditional kochujang is made using fermented cooked soybeans (meju), red pepper powder, and glutinous rice. Fermentation is carried out in an open place and is conducted by bacteria and yeasts contained in the meju. The commercial ones are produced using koji, steamed rice with a starter culture (*A. oryzae*). The traditional product is ready to eat after six months of fermentation, while the commercial product is fermented only for 2 to 4 weeks [57,115,199]. Kochujang has a characteristic taste. It is sweet because of the hydrolysis of rice starch during fermentation, spicy due to the addition of red pepper, and savory due to the hydrolysis of soybean protein [115].

##### Nutrients and Phytzocompounds

Kochujang contains 8.3–19.3 g of protein, 1.1 g of lipids, 43.8 g of carbohydrates, and 14.6 g of fiber per 100 g [200]. Kochujang contains a substantial amount of salt (2.4 g/100 g) [201]. Similar to previously described fermented soybean products, kochujang contains isoflavones, though their content is more than 30 times lower than in CGJ [202]. Due to the addition of pepper, kochujang is a dietary source of capsaicin (Table 1). Capsaicin activates transient receptor potential vanilloid channel 1, which subsequently activates metabolic modulators, including *PPAR-α*, the glucagon-like peptide 1 gene (*GLP-1*), the uncoupling protein 1 gene (*UCP1*), and *AMPK* [203].

##### Microbiota

The method of preparation of kochujang in different regions has a significant impact on the types of microorganisms present in the product. *Bacillus* is the most abundant bacterial genus in kochujang [58,59]. Some *Bacillus* strains isolated from kochujang have anti-radical properties, SOD-like activity, and ACE inhibitory potential. The most effective ACE inhibitors were reported to be the *B. amyloliquefaciens* and *B. velezensis* strains [58]. *B. amyloliquefaciens* and one of the *B. subtilis* strains isolated from kochujang had bile salt tolerance, acid tolerance, colon cell adhesion ability, and a lack of *B. cereus*-related endotoxins expression. Hence, they could be considered as potential probiotic strains [58].

Although Park et al. [59] reported that LAB are not present in kochujang, Nam et al. [199] and Ha et al. [58] identified several LAB genera, including *Lactobacillus*, *Tetragenococcus*, and *Weissella* [199]. The dominant yeast species is *Zygosaccharomyces pseudorouxii*, and the most abundant fungus is *Aspergillus oryzae* (Table 1) [59].

##### The Relevance of Kochujang in the Context of MetS

Kochujang may be beneficial for patients with MetS due to its antioxidant [117], anti-obesity [57,115,117,118,204], hypolipemic [57,115,117,118], and anti-diabetic properties (Table 2) [115,116]. Some bioactive compounds, including *p*-coumaric acid, N6,N6,N6-trimethyllysine, and methionine, seem to be responsible for the anti-obesity effect [205]. In 3T3-L1 cells, inhibition of lipid accumulation, reduced adipocyte size, and leptin secretion after kochujang extract administration seem to be mediated by inhibition of *PPAR-γ* and *SREBP-1c* expression. Additionally, enhanced expression of hormone-sensitive lipase (HSL) stimulates lipolysis [204]. In an in vivo model, kochujang reduced body fat deposition by enhancing the expression of hepatic lipolysis enzymes (*CPT-1* and *ACS*) and inhibiting lipolytic enzyme (*ACC*) expression. Anti-obesity action may also be supported by the upregulated expression of *UCP-1*, a gene associated with thermogenesis [115].

Kochujang acts as an *α*-glucosidase inhibitor in vitro [205]. In pancreatectomized rats, kochujang modulates glucose homeostasis by improving hepatic insulin sensitivity, not by promoting *β*-cell function. Moreover, kochujang reduced TG liver accumulation by enhancing STAT3-AMPK signaling, which reduced liver TG accumulation caused by the activation of AMPK [116]. The hypolipidemic effect may be due to the synergistic or additive effect of capsaicin and isoflavones in lipid metabolism [57].

#### 6.1.5. Doenjang

Doenjang is a traditional Korean soybean salty and alkaline paste. In Korean cuisine, it is a basis for soups, stews, and salad dressings [19,55]. In the homemade process, doenjang is made by fermenting meju in water and salt, while the commercial product is made with koji (fermented rice), soybeans, and water, which shortens the fermentation period [206]. A characteristic feature of doenjang is its high salt content, which reaches up to 12% [55].

##### Nutrients and Phytocompounds

Traditionally, fermented doenjang is characterized by a higher amount of polyphenols than the soybean paste fermented with *A. oryzae* starter culture (omitting the traditional procedure): 382.08 vs. 226.85 mg/100 g [120]. This may be due to a lower *Bacillus* cell count and lower diversity in *Bacillus* species in the product fermented with the starter culture. Traditionally, fermented doenjang contains mainly isoflavone aglycones, in contrast to commercial doenjang, where isoflavones such as glycosides and maloylglycosides occur [206]. Similar to the soy product described above, doenjang contains free amino acids, biogenic amines (histamine and tyramine), pyrazines, FA, GABA, furfural, and a variety of volatile compounds [56,207]. Moreover, there is some evidence that selected *B. subtilis*, *B. amyloliquefacience*, and *B. frigoritolerance* strains isolated from doenjang produce a variety of volatile compounds, including peptides (di- and oligopeptides), volatile compounds (butanodienone, 3-methylbutanal, 3-hydroxy-2-butanone), and pyrazines, which affect the sensory characteristics of the product (Table 1) [208].

##### Microbiota

The two main bacterial orders in doenjang are *Bacillales* (85.93%), followed by *Lactobacillales* (13.58%) [55]. The bacterial community consists of genera such as *Longilactobacillus*, *Tetragenococcus*, *Bacillus*, and *Leuconostoc* [209]. According to Mun et al. [55], the predominant species is *Bacillus paralicheniformis*, which represents over 69% of the bacterial community in doenjang, while Lee et al. [206] reported that the main bacterial species in doenjang are *Bacillus amyloliquefaciens*, *B. amyloliquefaciens* subsp. *plantarum*, *Bacillus licheniformis*, and *Bacillus subtilis*. The dominant genus in the fungal community is *Debaryomyces*, followed by *Wickerhamomyces* and *Mucor* [209]. Animal treatment with a mixture of *Bacillus* originating from doenjang resulted in reduced body mass, reduced subcutaneous fat mass, and improved glucose tolerance [120]. The characteristics of the microbial community of doenjang are presented in Table 1.

##### The Relevance of Doenjang in the Context of MetS

It seems that doenjang may be beneficial in MetS. To date, it has been demonstrated that doenjang possesses antioxidant [206], anti-inflammatory [119], anti-obesity [60,119,120,121,122], hypotensive [55,121], anti-diabetic [120,121], and hypolipidemic properties (Table 2) [119,120,122].

In adipose tissue, doenjang affects the expression of some oxidative stress markers (heme-oxygenase-1 and p40phox) and inflammatory markers (tumor necrosis factor-*α* (TNF-*α*), monocyte chemoattractant protein-1 (MCP-1), and macrophage markers (CD68 and CD11c). This suggests that the biologically active compounds produced during fermentation and maturation are responsible for antioxidative and anti-inflammatory action [60]. The anti-obesity properties of doenjang are a result of its impact on leptin and adiponectin levels [60,120]. Doenjang could also promote hypotrophy and hypoplasia of adipocytes [121] and reduce the amount of crown-like structures, a pathological histological structure occurring in fatty tissue that consists of necrotic adipocytes surrounded by aggregated macrophages [60].

Although doenjang contains a high amount of salt, it seems to be beneficial in hypertension. In 3T3-L1 cells treated with salt and doenjang, a reduction in *PPAR-γ*, *AGT*, and *Ace* expression was observed compared to control cells. Doenjang was found to reduce *PPAR-γ* expression to a similar level as losartan (10^−4^ M) and to a greater level than captopril (10^−4^ M). Doenjang reduced Ace more effectively than losartan (10^−4^ M) and was comparable to captopril (10^−4^ M) [121]. A rodent model study conducted by Mun et al. [55] revealed that doenjang supplementation was associated with a reduction in serum renin levels. Additionally, the mRNA expression of *Ace*, angiotensin type 1 receptor, mineralocorticoid receptor, and Na^+^/Ca^+^ exchanger in the kidney cortex of doenjang-supplemented animals was significantly lower than in animals on a high-salt diet without additional supplementation [55].

In vitro and in vivo evidence indicates that doenjang acts as an *α*-glucosidase inhibitor [56,205] and affects insulin sensitivity through specific molecular mechanisms [120]. Long-fermented doenjang improved insulin sensitivity in skeletal muscle and liver. Additionally, doenjang suppresses the expression of genes related to inflammation in the liver (*TNF-α*, *INFγ*, *MCP-1*, *IL-1β*, and *IL-6*) and skeletal muscle (*TNF-α*, *INFγ*, and *IL-1β*). In skeletal muscle, doenjang enhanced AMPK phosphorylation [120]. Additionally, the reduction in plasma TNF-*α* and resistin levels may be linked with a hypolipidemic effect under HFD conditions [119]. Cha and colleagues [122] confirmed a lipid-lowering effect in humans. They suggested that this effect can be attributed to the action of isoflavones.

### 6.2. Other Examples of Fermented Legumes

Although the most popular fermented legume is soybean, other fermented legumes are also of interest. The fermentation of beans, fava beans, or lentils may be beneficial in terms of creating new functional foods. Since literature reports on various fermented legumes are limited, these products will be characterized briefly, excluding the precise characteristics of the microorganisms and bioactive compounds.

Similar to soybeans, the breakdown of proteins into bioactive peptides occurs during the fermentation of other legume seeds. Both the peptides present in *L. plantarum* 299v fermented fava bean, as well as the peptides present in *B. subtilis* fermented lentils, reveal antioxidant and ACE inhibitory properties in vitro [210,211].

In addition, fermented mung beans could be considered a functional food in preventing MetS. In diabetic mice, a fermented mung bean extract improved glucose levels and the lipid profile [212], while in hypercholesterolemic rodents, mung bean extract reduced serum TC, TG, and LDL and improved the level of HDL. Moreover, the extract enhanced the expression of apolipoprotein-E (ApoE) and downregulated the expression of neuropeptide Y (Npy), which was found to be associated with an increased appetite and lower energy expenditure in obese rodents [213]. The hypolipemic effect was also shown by fermented red beans. An extract prepared from *B. subtilis* fermented red bean improved the serum lipid levels, as well as liver TC content and PPAR-α protein [214].

## 7. Microbial Metabolites

**Figure 2:**
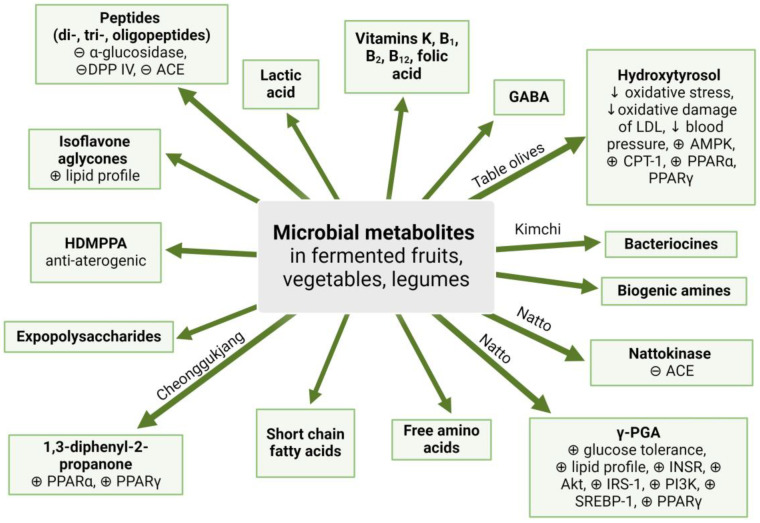
Microbial metabolites in fermented fruits, vegetables, and legumes. ↓, decrease; ⊕, improvement; ⊖, inhibition; ACE, angiotensin-converting enzyme; Akt, protein kinase B; AMPK, AMP-activated protein kinase; CPT-1, carnitine palmitoyltransferase 1; DPP IV, dipeptidyl peptidase 4; GABA, *γ*-aminobutyric acid; HDMPPA, 3-(40-hydroxyl-30,50-dimethoxyphenyl) propionic acid; PI3K, phosphatidylinositol 3-kinase; PPAR, peroxisome proliferator-activated receptor; SREBP-1, sterol regulatory element-binding protein 2; *γ*-PGA, *γ*-polyglutamic acid.

The health-promoting properties of fermented products may be attributed to the formation of novel compounds that were not present in raw ingredients before fermentation. Research suggests that fermented products may have a stronger effect on the parameters related to metabolism than raw ingredients [86,197,213]. The beneficial effects on MetS components are attributed to metabolites that include free amino acids (methionine, ornithine), peptides (di- and oligopeptides), exopolysaccharides, polyphenols (tyrosol, HT), bacterial enzymes (NK), and isoflavone aglycones, characteristic for fermented soybean products. The bioactive compounds in fermented plant foods are summarized in Figure 2.

## 8. New Trends in Fermented Foods

During the fermentation process, various modifications can be applied to obtain products with more favorable sensory characteristics or health-promoting properties. Using starter strains can provide great opportunities to obtain products with improved quality and desirable properties. Fermented plant products can be fermented with probiotic starter strains and, in consequence, used as a carrier for the probiotic bacteria. Previously, the products used for this purpose were dairy-based and unavailable to people with lactose or milk protein intolerance [215].

Due to the high salt content of most fermented products, a noteworthy research trend is the development of products with a reduced salt content. To maintain favorable sensory characteristics with a reduced salt content, the addition of a starter culture may be beneficial. An example of a product that could be considered in this category is low-sodium olives produced using a *Lactobacillus plantarum* F3. 3 starter strain [23,215]. In some cases, strains can produce some beneficial constituents or degrade undesirable compounds. For example, *Levilactobacillus brevis*, *Lactiplantibacillus* plantarum, or *Enterococcus faecium* strains could be applied to increase the GABA content in kimchi [124], or selected *Lactiplantibacillus* plantarum strains can be applied in olive production due to their oleuropeinolytic activity [216].

An alternative way to obtain products with health benefits may be an addition of herbs [124], plant extracts [217], and condiments that are not normally used in a certain product [218]. An example of an alternative product is kimchi with a variety of ingredients, including mushrooms, pear, Chinese pepper, sea tangle juice, green tea, mistletoe extract, and probiotic strains [16,131], or table olives made with a polyphenol-rich olive leaf extract [217].

## 9. Conclusions and Future Perspectives

Fermented plant-based foods can enrich the diet with probiotics and biologically active compounds, both those contained in plants and those originating from microbial metabolism. This review provides insights into the potential use of fermented fruits, vegetables, and legumes as a functional food and source of probiotic strains and biologically active compounds, which can be applied in the prevention and alleviation of metabolic disturbances. Fermented plant-based foods are a dietary source of bioactive peptides, free amino acids, short-chain fatty acids, GABA, and exopolysaccharides. Fermented soybean products, due to the high content of isoflavone aglycones, may be a superfood used in improving lipid profiles. Natto, which contains *γ*-PGA, is a well-studied product that may offer multiple benefits when included in the diet of individuals with MetS. *γ*-PGA contributes to the regulation of metabolism by modulating the expression of PPAR-*γ*, INSR, and Akt. In the context of metabolic disorders, olives represent another potentially beneficial dietary component. Hydroxytyrosol, produced from oleuropein as a result of lactic acid fermentation, has been shown to support blood pressure regulation and influence the expression of key metabolic regulators, including AMPK, PPAR-*α*, and PPAR-*γ*.

Some products, such as kimchi and fermented soybeans, mostly characteristic of Asian countries, have been extensively studied in terms of their impact on metabolic health (in both animal and human models). Despite the presence of certain bacterial strains and bioactive compounds in European fermented foods (table olives or sauerkraut), which are proven to be beneficial to metabolic health, there are no studies on the impact of these foods in general on metabolic parameters.

Globalization has led to the growing popularity of certain fermented products that were previously available only in the regions where they were traditionally produced. Fermented products made from soy or cabbage are considered relatively easy to produce in various parts of the world, due to the low cost of raw materials and processing, as well as the availability of specific starter cultures. Fermented foods are gaining popularity among younger consumers. The consumption of fermented products among younger individuals is driven not only by curiosity toward novel foods but also by increasing public awareness of their potential health benefits. Examples of products originating from other regions that have been well accepted by consumers include kimchi and fermented soy products (such as tempeh), which are now available on European markets. It is noteworthy that the scientific literature rarely discusses potential adverse effects or contraindications associated with the consumption of fermented plant-based foods. The most commonly cited contraindication to consuming fermented products is hypertension. This contraindication is not clear-cut, as some studies have reported the ‘salt paradox’—a phenomenon in which fermented products exert a hypotensive effect despite containing high levels of salt.

The primary challenges in the production of plant-derived fermented foods are to obtain products with consistent, reproducible quality, low in anti-nutritional compounds, and high in health-promoting constituents and probiotic microorganisms. It is also crucial to consider the sensory characteristics of fermented products and to formulate recommendations regarding appropriate portions of the products. Furthermore, studies on the development of dietary supplements, based on freeze-dried fermented foods and extracts, could expand their application.

## Figures and Tables

**Table 1:** Characteristics of microbial communities and main biologically active compounds in fermented fruits, vegetables, and legumes.

Product	Raw Ingredient	Characteristic Microorganisms	Main Bioactive Compounds	Country/Region of Origin	References
Table olives	Olive fruits	**Bacteria:** *L. acidipiscis*, *L. brevis*, *L. casei*, *L. coryniformis*, *L. fermentum*, *L. helveticus*, *L. paracasei*, *L. parafarraginis*, *L. paraplantarum*, *L. pentosus*, *L. plantarum*, *L. rhamnosus*, *E. faecalis*, *E. faecium*, *Lc. formosensis*, *Lc. lactis*, *Leu. mesenteroides*, *Ped. acidilactici*, *Ped. damnosus*, *S. inulinus*, *S. terrae*, *St. thermophilus*, *W. paramesenteroides*, *W. hellenica* **Yeasts:** *Wickerhamomyces anomalus*, *Wickerhamomyces sydowiorum*, *Saccharomyces cerevisiae*, *Pichia kluyveri*, *Pichia membranifaciens*, *Brettanomyces custersianus*, *Candida orthopsilosis*, *Candida tropicalis*, *Debaryomyces hansenii*	**Phenolic compounds:** hydroxytyrosol, tyrosol, rutin, luteolin, cyanidin, and delphinidin glucosides (only in black olives) **Iridoids:** oleuropein, verbascoside **Organic acids:** lactic acid **SCFAs:** acetic acid, butyric acid **Triterpenic acids:** maslinic acid, oleanolic acid **MUFA:** Oleic acid **Sterols:** *β*-sitosterol, Δ5-avenasterol	Mediterranean countries (Spain, Portugal, Greece, and Italy)	[23,27,28,29,30]
Caper	Caper berries	**Bacteria:** *L. plantarum*, *L. Paraplantarum*, *L. Pentosus*, *L. Fermentum*, *L. Brevis*, *Ped. Pentosaceus*, *Ped. Acidilactici*, *E. faecium* **Yeasts:** *Aureobasidium pullulans*	**Phenolic compounds:** quercetin, quercetin glycosides (mainly rutin), kaempferol, kaempferol glycosides, isorhamnetin, isorhamnetin glycosides, myricetin, ferulic acid, vanillic acid, epicatechin	Mediterranean countries	[31,32,33]
Kimchi	Chinese cabbage with other vegetables and condiments	**Bacteria:** *Leu. mesenteroides*, *Leu. gasicomitatum*, *Leu. gasicomitatum*, *Leu. kimchii*, *Leu. miyukkimchii*, *L. brevis*, *L. plantarum*, *L. kimchii*, *L. kimchiensis*, *L. koreensis*, *W. koreensis*, *Lc. kimchii*, *Tetragenococcus* spp., *St. faecalis*, *Ped. cerevisiae*, *Bacillus* spp. **Yeasts:** *Lodderomyces* spp., *Candida* spp., *Trichosporon* spp., *Saccharomyces* spp., *Pichia* spp.	**Sulfur compounds:** S-methlycysteinsulfoxide, S-allylcysteinsulfoxide **Sterols:** *α*-sitosterol **Amino acids:** ornithine **Others:** capsaicin, HDMPPA, gingerol	Korea	[34,35,36]
Sauerkraut/ Fermented cabbage	Cabbage	**Bacteria:** *L. plantarum*, *Leu. mesenteroides*, *Ped. pentosaceus*, *Levilactobacillus* spp., *Paucilactobacillus* spp., *Secundilactobacillus* spp. **Yeasts:** *Cryptococcus macerans*, *Debaryomyces hansenii*, *Pichia fermentans*, *Wickerhamomyces anomalus*, *Rhodotorula mucilaginosa*	**Organic acids:** lactic acid, malic acid **SCFAs:** acetic acid, propionic acid, butyric acid **Biogenic amines:** tyramine, putrescine **Amino acids:** alanine, leucine **Exopolysaccharides** **Esters:** ethyl acetate, ethyl lactate **Pyrazines:** 2,5-dimethylpyrazine **Sulfur compounds:** allyl isothiocyanate, dimethyl sulfide **Terpenes:** geranyl acetone **Others:** dimethyl sulfoxide, uracil, ascorbinogen	Europe, USA, China	[37,38,39,40,41,42]
Natto	Soybean	*Bacillus subtilis* var. natto	**Peptides and amino acids** **Exopolysaccharides:** levan **Isoflavones:** daidzein, genistein, glycitein, and corresponding *β*-glucosides **Ketones:** acetoin **Pyrazines:** di, tri, tetramethyl pyrazines **Biogenic amines:** spermine, followed by spermidine, tyramine **SCFAs:** acetic acid, isobutyric acid, isovaleric acid **Others:** *γ*-PGA, phenol, 2-metoxyphenol	China, Japan	[43]
Tempeh	Soybean	**Bacteria:** *E. cecorum*, *L. agilis*, *L. fermentum*, *L. mucosae*, *L. delbrucki*, *Acetobacter indonesiensis*, *Weisella* spp., *Enterococcus* spp., *Leuconostoc* spp., *Paenibacillus* spp., *Bacillus* spp. **Yeasts:** *Pichia guilliermondii*, *Candida tropicalis*, *Pichia norvegensis*, *Sporopachydermia lactativora*, *Trichosporon asahii* **Molds:** *R. microsporus*, *R. delemar*, *R. oligosporus*, *R. oryzae*, *R. stolonifer*, *Mucor* spp., *Rhizomucor* spp.	**Isoflavones:** daidzein, genistein, glycitein, and corresponding *β*-glucosides **Peptides and amino acids** **SCFAs** **Others:** GABA	Indonesia (also introduced in Japan, India, Europe, and Africa)	[44,45,46,47,48,49,50]
Cheonggukjang	Soybean	**Bacteria:** *B. piscis*, *B. coagulans*, *B*. *carboniphilus*, *B. hisashii*, *Ab. aneurinilyticus*, *S. equorum*, *B. subtilis*, *B. licheniformis*, *B. amylolquefaciens*, *B. haynessi*, *B. velazensis*	**Biogenic amines:** tyramine, *β*-phenylethylamine, putrescine **Saponins:** soyasapogenol A and B **Ketones:** 1,3-diphenyl-2-propanone **Organic acids:** lactic acid, 2-hydroxyglutaric acid **Pyrazines** **Aromatic compounds:** 4 -(nonafluoro-tert-butyl) nitrobenzene **Isoflavones:** daidzein, genistein, glycitein, and corresponding *β*-glucosides **Others:** GABA, *γ*-PGA	Korea	[51,52,53,54]
Doenjang	Soybean	**Bacteria:** *B. paralicheniformis*, *B. subtilis*, *B. acidicola*, *B. dabaoshanensis*, *B. idriensis*, *B. carboniphilus*, *B. aerius*, *B. crescens*, *E. hirae*, *E. phoeniculicola*, *Ped. acidilacti*, *E. faecalis*, *Ped. claussenii*, *B. licheniformis*, *B. athrophaeus*, *Enterobacter soli*, *Lentibacillus* sp., *Enterobacter* sp. **Yeasts:** *Zygosaccharomyces pseudorouxii*, *Zygosaccharomyces mellis*, *Candida versatilis*, *Ogatea polymorpha*, *Saccharomyces cerevisiae*	**Isoflavones:** daidzein, genistein, glycitein, and corresponding *β*-glucosides **Amino acids:** glutamic acid **Fatty acids:** isovaleric acid, **Volatile compounds:** 3-methyl butanal, benzeneacetaldehyde, *α*-curcumene, *β*-sesquiphellandrene, diallyl disulfide **Pyrazines** **Others:** GABA	Korea	[55,56]
Kochujang	Soybean	**Bacteria:** *B. amyloquefaciens*, *B. carboniphilus*, *B. circulans*, *B. coagulans*, *B. lentus*, *B. licheniformis*, *B. megaterium*, *B. pumilus*, *B. stearothermophilus*, *B. sonorensis*, *B. subtilis*, *B. thuringiensis*, *Aneurinibacillus thermoaerophilus*, *Brevibacillus borstelensis*, *E. faecalis*, *E. faecium*, *L. delbrucki*, *L. fermentum*, *L. fructivorans*, *L. gassei*, *L. halophilus*, *L. plantarum*, *L. salivarius*, *L. sakei*, *L. paracasei*, *W. confuse* **Yeasts:** *Saccharomyces cerevisiae*, *Zygosaccharomyces rouxii*	**Isoflavones** **Others:** capsaicin	Korea	[57,58,59,60]
**Conclusions:** The microbial communities of table olives, capers, kimchi, and sauerkraut consist mostly of LAB, including *L. plantarum* and *Pediococcus* spp., as well as yeasts. In fermented soybean products, mostly *Bacillus* species have been identified. In tempeh, molds such as *Rhizopus* spp. and *Mucor* spp. have also been identified. The most frequently identified groups of bioactive compounds in fermented foods include organic acids, SCFAs, GABA, free amino acids, and biogenic amines. Olives and capers are dietary sources of phenolic compounds, primarily flavonoids. A characteristic group of compounds found exclusively in table olives are iridoids. Kimchi and sauerkraut are produced from fermented cabbage and, as a result, are rich in sulfur compounds. Fermented soybean products typically contain isoflavone aglycones (daidzein, genistein, glycitein), peptides, and amino acids. A unique and health-promoting compound, *γ*-PGA, has been reported in natto and cheonggukjang.					

*γ*-PGA, *γ*-polyglutamic acid; GABA, *γ*-aminobutyric acid; SCFAs, short-chain fatty acids.

**Table 2:** Effects of fermented fruits, vegetables, and legumes on parameters related to metabolic syndrome.

Product	*n*	Animals/ Patients	Length of Study	Intervention	Control	Health-Promoting Effect ^1^	References
Caper fruit pickle	44	Subjects with a BMI of 25–35 kg/m^2^ and NAFLD	12 weeks	Caper fruit pickle (40–50 g/day) and consultation with a nutritionist	Consultation with a nutritionist	**Anti-obesity effect:** ↓ BW, ↓ BMI (compared to baseline) **No hypolipidemic effect:** Ø TC, Ø TG, Ø LDL, Ø HDL	[77]
Caper fruit pickle	44	Subjects with a BMI of 25–35 kg/m^2^ and NAFLD	12 weeks	Caper fruit pickle (40–50 g/day) and consultation with a nutritionist	Consultation with a nutritionist	**No anti-inflammatory effect:** Ø hs-CRP **Anti-obesity effect:** ↓ waist circumference **Hypolipidemic effect:** ↓ LDL/HDL, ↓TG/HDL, ↓ TC/HDL (compared to baseline) **No anti-diabetic effect:** Ø insulin, Ø HOMA-IR	[82]
Caper fruit pickle	60	Subjects newly diagnosed with hyperlipidemia and prescribed low-dose atorvastatin	8 weeks	Caper fruit pickle (40–50 g/day) and 10 mg atorvastatin	10 mg atorvastatin	**Hypolipidemic effect:** ↓ TC, ↓ LDL, ↓ TG, ↑ HDL	[83]
Kimchi	22	Obese and overweight subjects (crossover study)	4 weeks	Kimchi (300 g/day)	Baseline	**Anti-inflammatory effect:** Ø CRP, Ø IL-6, Ø TNF-*α*, ↓ MCP-1 **Anti-obesity effect:** ↓ BW, ↓ BMI, ↓ WHR, ↓ body fat, Ø adiponectin, ↓ leptin **Anti-diabetic effect:** ↓ FBG, ↓ FBI, **No hypotensive effect:** Ø systolic BP, Ø diastolic BP **Hypolipidemic effect:** ↓ TC, Ø LDL, Ø HDL, Ø TG	[84]
Kimchi fermented with *Weissella koreensis* OK1-6	7	Obese mice	12 weeks	HFD with kimchi powder (3%)	HFD	**Anti-obesity effect:** ↓ BW, ↓ epididymal adipose tissue, ↓ leptin **Anti-diabetic effect:** ↓ insulin **Hypolipidemic effect:** ↓ TC, Ø TG	[85]
Kimchi	21	Prediabetic subjects (crossover study)	8 weeks	Kimchi (300 g/day)	Baseline	**No anti-inflammatory effect:** Ø IL-1*β*, Ø IL-6, Ø IL-10, Ø TNF-*α*, Ø MCP-1, Ø CRP, Ø FGF-21 **Anti-obesity effect:** ↓ BW, ↓ BMI, ↓ WC, ↓ body fat (%, kg), Ø adiponectin **Anti-diabetic effect:** ↓ HbA1c, Ø FBG, ↓ FBI, ↓ HOMA-IR, ↑ Matsuda Index, ↑ QUICKI, ↑ DI, Ø IGI **Hypotensive effect:** ↓ systolic BP, ↓ diastolic BP	[86]
Kimchi	100	Healthy subjects	1 week	Kimchi (210 g/day)	Kimchi (15 g/day)	**Antioxidative effect:** ↑ TAC (statistically significant in both groups, but not between groups) **Anti-diabetic effect:** ↓ FBG (statistically significant in both groups and between groups) **Hypolipidemic effect:** ↓ TC, ↓ LDL, ↓ TG (statistically significant in both groups, but not between groups)	[36]
Kimchi	28	Healthy subjects	4 weeks	Standardized or functional kimchi with additional condiments and probiotics (210 g/day)	Baseline	**Anti-inflammatory effect:** ↓ IL-6, Ø hs- CRP, Ø MCP-1, Ø TNF-*α* (both standardized and functional kimchi) **Anti-obesity effect:** ↓ body fat (%), ↑ skeletal muscle mass, ↑ adiponectin, Ø BW, Ø WHR, Ø BMI, Ø body fat (kg), Ø leptin (statistically significant only in functional kimchi group) **Hypolipidemic effect:** ↓ LDL, ↑ HDL (both groups), ↓ TG, ↓ TC (only in functional kimchi group)	[16]
Cheonggukjang (CGJ)	100	90% of pancreatectomized diabetic rats	8 weeks	Soybean or CGJ (amount not given)	Diabetic rats, diabetic rats with rosiglitazone, non-diabetic rats	**No anti-obesity effect:** Ø BW, Ø food intake **Anti-diabetic effect:** ↓ glucose, ↓ insulin, improved OGTT, Ø *β* cell area (CGJ and soybean groups), ↓ *β* cell size and mass (only CGJ group), Ø *β* cells apoptosis	[87]
Cheonggukjang	30	C57BL/KsJ-db/db mice	6 weeks	Diet containing CGJ (5 g/100 g)	Diabetic mice, diabetic mice with rosiglitazone	**Anti-obesity effect:** ↓ BW, ↓ weight gain, ↓ food efficiency ratio, Ø food intake **Anti-diabetic effect:** ↓ glucose, ↓ HbA1c, Ø leptin, Ø insulin, ↓ glucagon, ↑ insulin (pancreatic tissue), Ø glucagon (pancreatic tissue)	[88]
Cheonggukjang	30	Mice	12 weeks	HFD with CGJ (40%)	Normal diet, HFD	**Anti-obesity effect:** ↑ food intake, ↓ weight gain, ↓ epididymal fat, ↓ back fat **Hypolipidemic effect:** ↓ TC, ↓ TG	[89]
Cheonggukjang	38	Obese mice	13 weeks	HFD with 30% cooked soybean powder or 30% CGJ powder	Normal diet, HFD	**Hypolipidemic effect:** Ø TG, ↓ TC (significant only for HFD compared to HFD + 30% CGJ powder)	[90]
Cheonggukjang	32	Rats	4 weeks	HFD with CGJ (20%) or CGJ made from soybean germinated under light or dark conditions (20%)	HFD	**No anti-obesity effect:** Ø BW, Ø food intake, Ø food efficiency ratio **Hypolipidemic effect:** Ø TC, ↓ LDL, ↓ TG, ↑ HDL (only for CGJ from soybean germinated under light conditions)	[91]
Cheonggukjang	80	Diabetic rats (induced by partial pancreatectomy)	8 weeks	HFD with cooked soybeans (10%) or traditional CGJ or CGJ fermented with a starter (*B. lichemiformis*) (10%)	Nondiabetic rats, diabetic rats	**Anti-obesity effect:** ↓ BW (only soybean), ↓ epididymal fat, ↓ caloric intake (all groups), ↓ leptin (only soybean) **Anti-diabetic effect:** ↓ FBG (all treatments), ↑ insulin, ↑ *β* cell area (both CGJ groups), ↓ *β* cell size (all groups), ↓ *β* cell apoptosis (all groups)	[92]
Cheonggukjang	87	Overweight/obese subjects (BMI ≥ 23 kg/m^2^ or WC ≥ 80 cm for women or ≥90 cm for men) (crossover study)	12 weeks	CGJ fermented with *B. licheniformis* (70 g/day)	Baseline, placebo	**Anti-inflammatory effect:** ↓ hsCRP (women, compared to baseline) **Anti-obesity effect:** Ø BW, Ø BMI, Ø body fat (kg), Ø lean body mass, Ø WC, Ø HC, ↓ WHR (significant only for women compared between groups), ↓ body fat (%) (significant only for women, compared to baseline and placebo) **Hypolipidemic effect:** ↑ TC (men, compared to baseline), Ø TC (women, compared to baseline), Ø LDL, ↑ TG (compared to baseline), Ø HDL, Ø FFA, ↑ ApoA1 (compared to baseline), ↓ ApoB (women, compared to baseline), ↓ ApoB/ApoA1 (compared to baseline)	[93]
Cheonggukjang	40	Obese mice	13 weeks	HFD with unfermented soybean (30%) or CGJ fermented with *γ*-PGA producing starter strain (*B. licheniformis*-67) (30%)	Normal diet and HFD	**Anti-obesity effect:** ↓ BW, ↓ weight gain, ↓ food efficiency ratio, ↓ leptin (significant compared to soybean and CGJ group), ↓ epidydymal fat (only CGJ group) **Anti-diabetic effect:** ↓ FBG, improved OGTT (only in CGJ group), Ø insulin **Hypolipidemic effect:** ↓TC (significant only for CGJ group), ↓ HDL, Ø TG (both soybean and CGJ groups)	[87]
Cheonggukjang	30	Obese mice	13 weeks	HFD with CGJ (10%)	Normal diet and HFD	**Anti-inflammatory effect:** ↓ MCP-1, ↓ TNF-*α* mRNA expression **Anti-obesity effect:** ↓ BW, ↓ weight gain, Ø food intake, ↓ food efficiency ratio, Ø epididymal fat, ↓ perirenal fat, ↓ leptin, ↑ adiponectin (all parameters significant compared to HFD, but leptin level also significant compared to baseline), ↓ adipocyte size **Anti-diabetic effect:** ↓ glucose, ↓ insulin **Hypolipidemic effect:** ↓ TC, ↓ TG	[94]
Cheonggukjang	55	Overweight/obese subjects with a BMI ≥ 25 kg/m^2^	12 weeks	Freeze-dried CGJ (26 g/day)	Baseline and placebo	**No anti-obesity effect:** Ø total fat, Ø visceral fat, Ø subcutaneous fat, Ø visceral subcutaneous ratio **Hypolipidemic effect:** Ø TC, Ø TG, Ø LDL, Ø HDL, Ø FFA, Ø ApoA1, ↓ ApoB (significant compared to baseline and placebo), Ø ApoB/ApoA1	[95]
Cheonggukjang	45	Subject with impaired fasting blood glucose	8 weeks	CGJ (20 g/day) or CGJ with red ginseng (20 g/day)	Placebo (starch 2 g/day)	**Antioxidative effect:** ↓ TBARS **Anti-diabetic effect:** ↓ FBG **Hypolipidemic:** ↓ TC, ↓ LDL, Ø HDL, Ø lipoprotein (a), ↓ Apo B/ApoA1 (only CGJ group)	[96]
Cheonggukjang	59	Obese subjects with a BMI ≥ 25 kg/m^2^	8 weeks	Freeze-dried traditional CGJ with a high microorganism content, with a low microorganism content, and commercial CGJ (3 g/day)	Baseline	**No anti-inflammatory effect:** Ø IL-6, Ø haptoglobin, Ø hs-CRP **No anti-obesity effect:** Ø HC, Ø WHR, Ø VF, Ø SF, Ø V/S **No hypolipidemic effect:** Ø TC, Ø LDL, Ø HDL **No anti-diabetic effect:** Ø glucose, Ø insulin, Ø HOMA-IR, Ø QUICKI (the same effect on each type of supplementation)	[97]
Tempeh	48	STZ-induced diabetic rats	14 weeks	STZ + HFD with cooked soybean or tempeh or tempeh fermented with probiotics (*L. plantarum* and *R. oligosporus*) (40 mg/kg BW/day)	Control diet, STZ + HFD or STZ + HFD + pioglitazone	**Anti-diabetic effect:** improved OGTT, ↓ AC glucose, ↓ HbA1C, ↓ insulin, ↓ HOMA-IR **Hypolipidemic effect:** ↓ TC, ↓TG, ↓ LDL, ↓ FFA, ↑ HDL	[98]
Tempeh	20	STZ-induced rats	30 days	Tempeh powder (200 mg/kg BW/day)	Nondiabetic rats, diabetic rats	**Anti-diabetic effect:** ↓ insulitis, no effect on the diameter of pancreatic Langerhans islets	[99]
Tempeh	18	Rats	3 weeks	High cholesterol diet and tempeh flour (0.95 g/100 g BW/day)	Standard diet, high cholesterol diet	**Antioxidative effect:** ↓ MDA **Anti-obesity effect:** ↓ BW, Ø food intake **Hypolipidemic effect:** ↓ TC, ↓ TG, ↑ HDL	[100]
Tempeh	30	STZ-induced rats	4 weeks	Tempeh or tempeh fermented with fermented cassava tuber extract in amounts that provide 15 or 30% of protein	Nondiabetic rats, diabetic rats	**Anti-diabetic effect:** ↓ FBG (statistically significant compared to diabetic control, effect was slightly stronger according to tempeh fermented with cassava extract)	[101]
Tempeh	30	STZ-induced diabetic mice	3 weeks	Tempeh (10, 20, or 40 mg/100 g BW/day)	Nondiabetic mice, diabetic mice, diabetic mice with metformin	**Anti-diabetic effect:** ↓ blood glucose **Hypolipidemic effect:** ↓ TC, ↓ LDL, ↓ TG, ↑ HDL	[102]
Tempeh fermented in both aerobic and anaerobic conditions	20	STZ-induced diabetic mice	3 weeks	Tempeh (10, 20, or 40 mg/100 g BW/day)	Nondiabetic mice, diabetic mice	**Anti-diabetic effect:** ↓ blood glucose, ↑ insulin, ↓ insulin secretion (HOMA-*β*) (statistically significant compared to diabetic control, glucose level was statistically significant also compared to baseline)	[103]
Tempeh	18	Db/db obese diabetic mice	12 weeks	Tempeh (300 mg/kg or 600 mg/kg)	Db/db obese diabetic mice	**Anti-obesity effect:** ↓ BW (for 600 mg/kg, compared to baseline and 300 mg/kg) **Anti-diabetic effect:** ↓ blood glucose **Hypolipidemic effect:** ↓ lipid accumulation in adipocytes (for 600 mg)	[104]
Tempeh	30	Obese rats	4 weeks	High-fat sucrose diet and freeze-dried tempeh fermented with *R. oligosporus* (60 mg/kg BW) or *R. oligosporus* and *L. rhamnosus* GG co-fermented tempeh in dose 60 mg/kg BW or 120 mg/kg BW	Standard diet, high-fat sucrose diet, and high-fat fructose diet with orlistat (120 mg/kg BW)	**Anti-inflammatory effect:** ↓ hsCRP (all interventions compared to negative control) **Anti-obesity effect:** ↓ BW (all interventions compared to negative control) **Hypolipidemic effect:** ↓ TC, ↓ LDL, ↓ TG, ↑ HDL (all interventions compared to negative control)	[105]
Tempeh gembus	41	Women with hyperlipidemia	2 weeks	Tempeh gembus (103 or 206 g/day) and nutrition education	Nutrition education, baseline	**Hypolipidemic effect:** ↓ TC, ↓ LDL (significant only compared to baseline, no between groups), Ø TG, Ø HDL	[106]
Tempeh	40	Obese women with BMI ≥ 25 kg/m^2^	4 weeks	Processed tempeh (150 g/day)	Control	**Anti-obesity effect:** ↓ WC, Ø BW, Ø BMI **No anti-diabetic effect:** Ø FBG	[107]
Tempeh	35	Patients with type 2 diabetes	12 weeks	Freeze-dried tempeh (2 g/day)	Baseline	**Hypolipidemic effect:** ↓ TG, Ø TC, Ø LDL, Ø HDL **Anti-diabetic effect:** ↓ HbA1c, Ø AC sugar	[108]
Tempeh	24	Subjects with TC ≥ 4.92 mmol/L	6 weeks	Tempeh (66 g/day)	Control group and baseline (n of subjects in control group = 3)	**Anti-obesity effect:** ↓ BW **Hypolipidemic effect:** ↓WC (statistically significant compared to baseline, no between groups); Ø fat (%), Ø visceral fat (%), Ø TC	[109]
Natto fractions	18	Rats	3 weeks	Diet containing 1% cholesterol and with 9% low-molecular-weight viscous substance from natto (LMWVS) or 9% soybean water extract (SWE) from soybean from natto	Diet with 1% cholesterol	**Ani-oxidative effect:** Ø Cu/Zn-SOD, Ø CAT, Ø GSH, ↓ TBARS, ↑ Mn-SOD (only LMWVS), ↑ GSH-Px (only SWE) **No anti-obesity effect:** Ø BW **Hypolipidemic effect:** Ø TC, Ø LDL, Ø HDL, Ø free cholesterol, Ø FFA, ↓ TG (both fractions)	[110]
Natto	50	Rats	4 weeks	Diet with 1% cholesterol and natto powder (750 mg/day or 1500 mg/day)	Diet, diet with 1% cholesterol, diet with 1% cholesterol, and aspirin (100 mg/day)	**No anti-obesity effect:** Ø BW **Hypolipidemic effect:** Ø TG, ↓ TC (only group with supplementation in dose 1500 mg/day)	[111]
Natto	30	Mice	4 weeks	HFD with natto (2.5% or 5%) or soybean (2.5% or 5%)	Control diet, HFD	**Antioxidative effect:** ↓ TBARS **Anti-obesity effect:** ↓ BW (natto 2.5%, natto 5%, soybean 5%), Ø food intake, ↓ total fat tissue **Anti-diabetic effect:** ↓ glucose (natto 5%, soybean 2.5%, soybean 5%), ↓ insulin (natto 5%), ↓ HOMA-IR (natto 2.5%) **Hypolipidemic effect:** Ø TG, ↓ TC (natto 2.5%, natto 5%, soybean 2.5%)	[112]
Natto	21	Mice	6 weeks	HFD with cooked soybean (15%) or tempeh (15%)	HFD	**No anti-obesity effect:** Ø BW, Ø visceral fat, Ø food intake **Anti-diabetic effect:** ↓ glucose (only the tempeh group compared to HFD) **Hypolipidemic effect:** Ø TC, Ø FFA, Ø HDL, ↓ TG (only tempeh group compared to HFD)	[113]
Natto	11	Overweight subjects with impaired glucose tolerance (crossover study)	2 weeks	Breakfast consisting of white rice, natto, okra, and Japanese yam	Breakfast consisted of white rice, boiled soybeans, potatoes, broccoli; baseline	**Antioxidative effect:** ↓ MDA-LDL (significant between groups) **No anti-obesity effect:** Ø BW, Ø adiponectin, Ø leptin **Anti-diabetic effect:** Ø HOMA-IR, Ø OGTT (glucose), Ø HbA1c, Ø 1,5-anhydroglucitol, Ø FBG, Ø FBI, ↓ hyperinsulinemia, ↑ CISI (statistically significant only compared to baseline, no between groups) **Hypolipidemic effect:** ↓ TC, ↓ LDL, Ø TG, Ø HDL	[114]
Kochujang (KJ)	30	Mice	12 weeks	HFD with KJ (22%)	Normal diet, HFD	**Anti-obesity effect:** ↓ BW, ↓ epididymal fat, ↓ back fat, ↑ food intake **Anti-diabetic effect:** ↓ glucose **Hypolipidemic effect:** ↓ TC, ↓ LDL, Ø HDL	[115]
Kochujang	60	90% of pancreatectomized diabetic rats	8 weeks	HFD with KJ (5%) fermented in a traditional method or a modern method (with *A. sojae*, *B. subtilis*)	Diabetic rats, non-diabetic rats	**Anti-obesity effect:** ↓ epididymal fat, ↓ leptin, ↑ caloric intake (both groups) **Anti-diabetic effect:** ↓ glucose, ↓ insulin, improved OGTT (both groups)	[116]
Kochujang	26	Hyperlipidemic subjects	12 weeks	Dried KJ fermented with *A. oryzae* (34.5 g/day)	Placebo, baseline	**Hypolipidemic effect:** ↓ TC (both compared to baseline and placebo), ↓ LDL (only compared to baseline), Ø HDL, Ø TG	[57]
Kochujang	53	Overweight or obese adults with BMI ≥ 23 kg/m^2^ or WHR ≥ 0.90 for males or ≥0.85 for females	12 weeks	Dried KJ (32 g/day)	Placebo, baseline	**Antioxidant effect:** Ø ORAC, Ø GSH, ↓ TRAP (compared to baseline), Ø SOD, ↓ CAT **No anti-obesity effect:** Ø BMI, Ø WHR, Ø WC, Ø visceral fat, Ø subcutaneous fat **No anti-diabetic effect:** Ø glucose, Ø insulin, Ø HOMA-IR, Ø HbA1c **Hypolipidemic effect:** Ø TC, Ø LDL, Ø HDL, Ø FFA, ↓ TG (compared to placebo), ↓ ApoA1, ↓ApoB (compared to baseline) **No hypotensive effect:** Ø SBP, Ø DBP	[117]
Kochujang	48	Overweight adults with BMI ≥ 23 kg/m^2^	6 weeks	Freeze-dried KJ (19 g/day): traditional with a high content of beneficial microorganisms (HTK), or traditional containing a low content of beneficial microorganisms (LTK), or a commercial KJ (CK)	Baseline	**No anti-inflammatory effect:** Ø hs-CRP **Anti-obesity effect:** ↓ hip circumference, ↓WHR (only CK group), ↓ visceral fat (only HTK group), ↓ WC (HTK and CK groups) **No anti-diabetic effect:** Ø adiponectin, Ø leptin, Ø subcutaneous fat, Ø BMI, Ø BW, Ø body fat (kg), body fat (%), Ø glucose **Hypolipidemic effect:** ↓ TC, ↓ LDL, ↓ TG, ↓ HDL (significant only for HTK)	[118]
Doenjang (DJ)	24	Mice	8 weeks	HFD with DJ (amount not given)	Normal diet, HFD	**Anti-inflammatory effect:** ↓ TNF-*α* **Anti-obesity effect:** ↓ BW, ↓ food efficiency ratio, Ø food intake, Ø epididymal fat, Ø leptin, Ø adiponectin **Hypolipidemic effect:** ↓ TG, ↓ FFA, Ø TC, Ø HDL	[119]
Doenjang	47	Mice	11 weeks	HFD with steamed soybeans (11.7%) or DJ (14.4%)	Low-fat diet, HFD	**Anti-obesity effect:** ↓ BW, ↓ epididymal fat, ↓ leptin (compared to HFD, only for DJ group, not in soybean group), Ø adipocyte size	[60]
Doenjang	28	Mice	14 weeks	HFD with short-term fermented soybean paste with starter (*A. oryzae*) (SFSP) or long-term fermented soybean paste (LFSP)	Normal diet, HFD	**Anti-obesity effect:** ↓ BW, ↓ subcutaneous fat (only LFSP group) **Anti-diabetic effect:** ↑ insulin sensitivity in liver and skeletal muscle (only LFSP group), Ø insulin **Hypolipidemic effect:** ↓ TG (only LFSP group), Ø TC	[120]
Doenjang	18	Rats	8 weeks	High-salt diet (8% NaCl) with freeze-dried DJ (water solution)	Normal diet, high-salt diet (8% NaCl)	**No anti-obesity effect:** Ø BW **Hypotensive effect:** ↓ SBP, ↓ serum renin	[55]
Doenjang	24	Rats	13 weeks	HFD with DJ (containing 8% of salt)	Normal diet and HFD, and HFD with 8% salt	**Anti-obesity effect:** ↓ BW, ↓ fat weight/BW, ↓ adipocyte size **Anti-diabetic effect:** ↓ glucose (lower than both the normal diet and HFD group) **Hypotensive effect:** ↓ SBP, ↓ serum aldosterone, Ø serum renin, Ø serum angiotensin II	[121]
Doenjang	51	Adults with BMI ≥ 23 kg/m^2^ or WHR ≥ 0.90 for males or ≥0.85 for females	12 weeks	Dried DJ (9.9 g/day)	Placebo and baseline	**Anti-obesity effect:** ↓ BW, ↓ body fat (kg), ↓ body fat (%), visceral fat (cm^2^) (compared both to baseline and placebo), ↓ WHR, ↓ total fat (cm^2^), ↓ subcutaneous fat (compared only to baseline) **Hypolipidemic effect:** Ø TG, Ø HDL, Ø FFA ↓ TC, ↓ LDL, ↓ ApoA1, ↓ ApoB (compared only to baseline)	[122]
**Conclusions:** Fermented caper fruits, kimchi, and DJ are fermented products that may be beneficial in reducing body weight. CGJ and natto appear to be the most well-studied products and are considered potential superfoods for patients with diabetes. Consumption of CGJ has been shown to reduce blood glucose levels, improve OGTT results, and positively affect *β*-cell function. Tempeh consumption has been associated with reductions in blood glucose levels and HbA1c in both human and animal models. The lipid profile may be improved following the consumption of fermented soybean products, including tempeh, natto, and KJ. To the best of our knowledge, there are no studies evaluating the effects of table olives and sauerkraut consumption on metabolic parameters in either animal or human models.							

^1^ Ø, no effect; ↓, decrease; ↑, increase; information about statistical significance is given in parentheses and refers to all the parameters listed earlier for a given health effect. AC sugar, fasting blood sugar; Apo A1, apolipoprotein A1; Apo B, apolipoprotein B; BW, body weight; CAT, catalase; CISI, Composite Insulin Sensitivity Index; DI, Disposition Index; FBG, fasting blood glucose; FBI, fasting blood insulin; HC, hip circumference; HFD, high-fat diet; IGI, insulinogenic index; MCP-1, monocyte chemoattractant protein-1; QUICKI, Quantitative Insulin Sensitivity Check Index; SF, subcutaneous fat; SOD, superoxide dismutase; TAC, total antioxidant status; TRAP, plasma total radical-trapping antioxidant; V/S, visceral fat/subcutaneous fat ratio; VF, visceral fat; WC, waist circumference; WHR, waist/hip ratio.

## Supplementary Materials

The following supporting information can be downloaded at: https://www.mdpi.com/article/10.3390/nu17121989/s1, Table S1: Effect of bacterial strains isolated from fermented vegetables and legumes on parameters related to metabolic syndrome.

## Abbreviations

The following abbreviations are used in this manuscript:

MetS	Metabolic syndrome
CVD	Cardiovascular disease
LAB	Lactic acid bacteria
GRAS	Generally Recognized as Safe
HT	Hydroxytyrosol
AMPK	AMP-activated protein kinase
ApoB	Apolipoprotein B
ACC	Acetyl-CoA carboxylase
DGAT	Diacylglycerol acyltransferase
HMGCR	3-Hydroxy-3-methyl-glutaryl-CoA reductase
FFA	Free fatty acids
HDMPPA	3-(40-Hydroxyl-30,50-dimethoxyphenyl) propionic acid
TG	Triglycerides
BP	Blood pressure
BMI	Body Mass Index
HOMA-IR	Homeostatic Model Assessment for Insulin Resistance
QUICKI	Quantitative Insulin Sensitivity Check Index
TLR 4	Toll-like receptor 4
NF-κB	Nuclear factor-κB
CGJ	Cheonggukjang
DJ	Doenjang
OGTT	Oral Glucose Tolerance Tests
DPP IV	Dipeptidyl peptidase 4
ACE	Angiotensin-converting enzyme
AX	Arabinoxylan
γ-PGA	γ-polyglutamic acid
NK	Nattokinase
HFD	High-fat diet
CEBP/α	CCAAT element binding protein α
HSL	Hormone-sensitive lipase
KJ	Kochujang
TNF-α	Tumor necrosis factor α
MCP-1	Monocyte chemoattractant protein-1
AGT	Angiotensinogen
LXRα	Liver X receptor alpha gene
SREBP-1c	Sterol regulatory element-binding protein 1c gene
SREBP-2	Sterol regulatory element-binding protein 2 gene
FAS	Fatty acid synthase gene
Ldlr	Low density lipoprotein receptor gene
Agpat5	1-Acyl-sn-glycerol-3-phosphate acyltransferase 5 gene
Acox2	Acyl-CoA oxidase gene
Akt	Protein kinase B
GLP-1	Glucagon-like peptide 1 gene
UCP1	Uncoupling protein 1 gene
Me	Malic enzyme
G6pdx	Glucose-6-phosphate dehydrogenase X-linked
Aco	Acyl-CoA oxidase gene
PEPCK	Phosphoenolpyruvate carboxykinase 1
