# Supplementary material for: Fermented Fruits, Vegetables, and Legumes in Metabolic Syndrome: From Traditional Use to Functional Foods and Medical Applications
Source: Nutrients. 2025 Jun 12;17(12):1989. doi: 10.3390/nu17121989 (PMC12195872; doi:10.3390/nu17121989)
Supplement: Supplementary file 1 [file nutrients-17-01989-s001.zip › nutrients-3658473-supplementary.pdf]

Table S1. Effect of bacterial strains isolated from fermented vegetables and legumes on parameters related to metabolic syndrome.

Strain and product	n	Animals/ patients	Length of study	Intervention	Control	Health-promoting effect[1]	References
L. brevis OPK-3 isolated from kimchi	10	Obese mice	12 weeks	HFD and orally administration of *L. brevis* OPK-3 (1 × 10^9 CFU/)	HFD	**Anti-inflammatory effect:** ↓ IL-1β, ↓ IL-6, ↓ TNF-α; ↓ expression of hepatic genes related to inflammation and immune response<br>**Anti-obesity effect:** ↓ BW, ↓ epididymal adipose tissue<br>**Anti-diabetic effect:** improved glucose tolerance (improved OGTT, ↓ glucose AUC)<br>**Hypolipidemic effect:** ↓ TC, ↓ TG, ↑ HDL<br>↓ hepatic TG, Ø hepatic TC, reduced lipid droplets infiltration in liver	(Park, Oh, and Cha 2020)
Lactobacillus sakei CJLS03 isolated from kimchi	114	Obese subjects with BMI ≥ 25 kg/m²	12 weeks	Orally administration of *Lactobacillus sakei* CJLS03 (5 × 10^9 CFU)	Placebo	**Anti-obesity effect:** ↓ body fat (kg) (statistically significant only between groups), ↓ waist circumference (statistically significant compared to baseline and between groups), Ø BW, Ø visceral fat<br>**No anti-diabetic effect:** Ø FBG, Ø HbA1c, Ø insulin, Ø HOMA-IR<br>**No hypolipidemic effect:** Ø TC, Ø TG, Ø HDL, Ø LDL, Ø ApoA1, Ø ApoB, ↓ FFA (statistically significant compared to baseline and between group)<br>**No hypotensive effect:** ↑ DBP (statistically significant between groups), Ø SBP	(Lim et al. 2020)
Lactobacillus plantarum HAC01 isolated from kimchi	40	Prediabetic subjects	8 weeks	Orally administration of *Lactobacillus plantarum* HAC01 (4 × 10^9 CFU/day)	Placebo	**Anti-diabetic effect:** ↓ 2h-PPG, ↓ HbA1c (statistically significant between groups), Ø FBG, insulin, HOMA-IR, QUICKI<br>**No hypolipidemic effect:** Ø TC, Ø HDL-C, Ø LDL, Ø TG, Ø adiponectin, Ø leptin	(Oh et al. 2021)

Lactobacillus plantarum S9 isolated from sauerkraut	30	HFD-induced MetS rats	6 weeks	Orally administration of *Lactobacillus plantarum* S9 (2 ml/day) *CFU not given	Normal diet, HFD diet	**Anti-obesity effect:** ↓ weight gain<br>**Anti-inflammatory effect:** ↓ LPS, ↓ TNF-α<br>**Anti-diabetic effect:** ↓ FBG, ↓ insulin, ↓ HOMA-IR, Ø HOMA-B<br>**Hypolipidemic effect:** ↓ TC, ↓ TG, ↓ LDL, ↑ HDL	(Zhao et al. 2022)
B. natto isolated from natto	42	C57BL/6J obese mouse	8 weeks	HFD with intragastric administration of *B. natto* in doses $1×10^7$, $1×10^8$, $1×10^9$ CFU/day	Low-fat diet without bacteria treatment, HFD without bacteria treatment, HFD with *L. plantarum* $1 × 10^8$ CFU	**Anti-inflammatory effect:** ↓ TNF-α (significant for all *B. natto* groups and *L. plantarum* group compared to HFD)<br>**Anti-obesity effect:** ↓ BW (significant for all *B. natto* groups compared to HFD), ↓ perirenal fat, ↓ epididymis fat, ↓ abdominal fat (significant for all *B. natto* groups and *L. plantarum* group)<br>**No anti-diabetic effect:** Ø FBG (but ↓ in *L. plantarum* group)<br>**Hypolipidemic effect:** ↓TC (significant for *B. natto* $1×10^7$, $1×10^8$ and *L. plantarum* group compared to HFD), ↓ TG (significant for all *B. natto* groups and *L. plantarum* group compared to HFD)	(Wang et al. 2020)
B. natto isolated from natto	48	Obese rats	9 weeks	HFD with intragastric administration of *B. natto* in doses $1×10^6$, $1×10^8$, $1×10^{10}$ CFU/mL/day	Low-fat diet without bacteria treatment, HFD without bacteria treatment, HFD with orlistat	**Anti-inflammatory effect:** ↓ TNF-α, ↓ IL-1β, ↓ IL-6, ↑ IL-6 ($1×10^{10}$ groups compared to HFD)<br>**Anti-obesity effect:** ↓ BW, ↓epididymal fat weight (all experimental groups compared to HFD)<br>**Anti-diabetic effect:** ↓ FBG, ↓ glucose AUC, ↓ fasting insulin (all experimental groups compared to HFD), ↓ HOMA-IR ($1×10^8$, $1×10^{10}$ groups compared to HFD)<br>**Hypolipidemic effect:** ↓ TC, ↓ LDL, ↓ TG, ↑ HDL (in all experimental groups compared to HFD)	(Sun et al. 2022)

[1]Ø, no effect; ↓, decrease; ↑, increase

AUC, area under the curve; BW, body weight; CFU, colony-forming unit; FBG, fasting blood glucose; HFD, high fat diet; MetS, metabolic syndrome; n, number of animals/participants
